# Experimental Pulmonary Tuberculosis in the Absence of Detectable Brain Infection Induces Neuroinflammation and Behavioural Abnormalities in Male BALB/c Mice

**DOI:** 10.3390/ijms21249483

**Published:** 2020-12-13

**Authors:** Jacqueline V. Lara-Espinosa, Ricardo A. Santana-Martínez, Perla D. Maldonado, Mario Zetter, Enrique Becerril-Villanueva, Gilberto Pérez-Sánchez, Lenin Pavón, Dulce Mata-Espinosa, Jorge Barrios-Payán, Manuel O. López-Torres, Brenda Marquina-Castillo, Rogelio Hernández-Pando

**Affiliations:** 1 Sección de Patología Experimental, Instituto Nacional de Ciencias Médicas y Nutrición Salvador Zubirán, CDMX 14080, Mexico; jvle_29031991@comunidad.unam.mx (J.V.L.-E.); zetter.salmon@gmail.com (M.Z.); dulmat@yahoo.com.mx (D.M.-E.); qcjbp77@yahoo.com.mx (J.B.-P.); lopeztorresmanuel88@gmail.com (M.O.L.-T.); 2 Laboratorio de Neuropatología Molecular, Instituto de Fisiología Celular, Universidad Nacional Autónoma de México, CDMX 04510, Mexico; rsantana@ifc.unam.mx; 3 Laboratorio de Patología Vascular Cerebral, Instituto Nacional de Neurología y Neurocirugía Manuel Velasco Suárez, CDMX 14269, Mexico; maldonado.perla@gmail.com; 4 Departamento de Psicoinmunologia, Instituto Nacional de Psiquiatria Ramón de la Fuente, CDMX 14370, Mexico; lusenbeve@yahoo.com (E.B.-V.); gipersanchez@hotmail.com (G.P.-S.); lkuriaki@imp.edu.mx (L.P.)

**Keywords:** *Mycobacterium tuberculosis*, neuroinflammation, behaviour abnormalities

## Abstract

Tuberculosis (TB) is a chronic infectious disease in which prolonged, non-resolutive inflammation of the lung may lead to metabolic and neuroendocrine dysfunction. Previous studies have reported that individuals coursing pulmonary TB experience cognitive or behavioural changes; however, the pathogenic substrate of such manifestations have remained unknown. Here, using a mouse model of progressive pulmonary TB, we report that, even in the absence of brain infection, TB is associated with marked increased synthesis of both inflammatory and anti-inflammatory cytokines in discrete brain areas such as the hypothalamus, the hippocampal formation and cerebellum accompanied by substantial changes in the synthesis of neurotransmitters. Moreover, histopathological findings of neurodegeneration and neuronal death were found as infection progressed with activation of p38, JNK and reduction in the BDNF levels. Finally, we perform behavioural analysis in infected mice throughout the infection, and our data show that the cytokine and neurochemical changes were associated with a marked onset of cognitive impairment as well as depressive- and anxiety-like behaviour. Altogether, our results suggest that besides pulmonary damage, TB is accompanied by an extensive neuroinflammatory and neurodegenerative state which explains some of the behavioural abnormalities found in TB patients.

## 1. Introduction

Tuberculosis (TB) is an infectious disease caused by *Mycobacterium tuberculosis* (*M. tb*) [1] and is considered a global priority due to the high number of new diagnoses and its 1.2 million deaths annually [2]. TB is associated with an increased risk of medical comorbidities contributing to a significant rise in mortality. Among the comorbidities related to TB, neuropsychiatric disorders are particularly concerning [3,4,5]. There is evidence that anxiety, depression and emotional distress are significant factors on the burden and disability of TB, and that they are strictly related to the severity and number of reported symptoms, higher rates of health services use, lower treatment compliance, a more extensive course of treatment, and reduced control of the disease and death [4]. Prevalence of depression and anxiety is higher in TB patients than in the general population [6,7,8,9].

In most cases, infection with *M. tb* is controlled effectively by the host’s immune system. However, when there is a failure in the immune response, the infection becomes active. Consequently, a series of mechanisms are activated to control the infection and eliminate the bacteria, which generates a chronic inflammatory state [10]. There is evidence for the role of pro-inflammatory cytokines in the development of neuropsychiatric symptoms. Pro-inflammatory cytokines such as interferon-gamma (IFNγ) and tumour necrosis factor-alpha (TNFα) might affect the progress of depressive disorder by regulating neuronal excitability, synaptic transmission, synaptic plasticity, excitotoxicity and neuronal survival [11]. These mechanisms generate brain inflammation which impacts established depression and pathophysiological anxiety processes, such as monoaminergic neurotransmission alteration, activation of Kynurenine Metabolism pathways, decrease in the synthesis of serotonin (5-HT) [12,13], hippocampal neuronal damage [14] and dysregulation of the hypothalamic–pituitary–adrenal (HPA) axis [15]. 

Furthermore, there are previous findings that suggest that acute and chronic inflammatory reactions such as obesity, chronic liver disease, inflammatory bowel disease, systemic administration of the Bacillus Calmette–Guerin (BCG) [16], infection with *M. lepraemurium* [17] and lipopolysaccharide (LPS) administration induce depressive-like behaviour autonomously of sickness behaviour. Both obesity and diabetes lead to increased neuroinflammation, essentially accelerating the ageing process [18]. In addition, it is well known that systemic inflammation contributes to increasing central inflammatory processes, principally in the hippocampus and hypothalamus [19]. 

Considering that *M. tb* induces chronic inflammation with substantial irregularities of cell-mediated immunity [20], we hypothesise that the peripheral inflammation generated during pulmonary TB infection could be affecting the central nervous system (CNS), independently of the presence of the bacilli in the brain, by activating and modifying the production of pro and anti-inflammatory cytokines within the brain that affect the monoaminergic neurotransmission and neuronal survival; this could be related to the development of neuropsychiatric disorders. 

Thereby, this study aimed to determine the effect of *M. tb* pulmonary infection in the CNS without brain infection. It has been previously reported that *M. tb* H37Rv shows minimal efficiency to infect the brain because of the CNS protection conferred by the blood–brain barrier (BBB) [21]. Thus, we used a model of progressive pulmonary TB in BALB/c mice infected with H37Rv to quantify pro and anti-inflammatory cytokines gene expression in the hypothalamus, hippocampus and cerebellum, and the disturbance in the levels of epinephrine, norepinephrine, serotonin and dopamine by high-performance liquid chromatography (HPLC) and histological damage. Different behavioural tests were performed during pulmonary TB. These tests included the study of sickness behaviour (locomotor activity, food intake and weight loss), anxiety-like behaviour, neurological severity score, short and long-term memory and depression-like behaviour. 

## 2. Results

### 2.1. There Is No Brain Infection during Progressive Pulmonary TB

**Figure 1:**
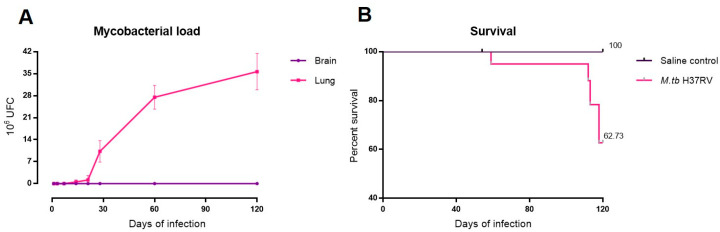
Mycobacterial loads in brain and lung and survival rate of BALB/c mice infected with 2.5 × 10^5^ colony-forming unit (CFU) of *M. tb* H37Rv. (**A**) Comparative quantification of CFU from lungs and brains of infected mice at each time of infection (*n* = 12). No bacilli loads were detected in the brain homogenates from mice infected with the H37Rv strain. (**B**) Survival rates of mice injected with saline solution (control) or infected with *M. tb* H37Rv (*M. tb* H37Rv) (*n* = 36). After two months of infection, infected mice started to die.

As we wanted to know if pulmonary infection without brain infection generates neuroinflammation, it was mandatory to confirm the lack of cultivable mycobacteria in the brain. In fact, brains collected from mice infected by the intratracheal route with *M. tb* H37Rv did not show bacillary growth in any day of infection, which confirms the previous report [21], while in the lungs there was a progressive increase in bacillary loads (quantification of colony-forming units (CFUs)) after 21 days of infection. After 2 months of infection, animals started to die in coincidence with high pulmonary bacillary burdens (Figure 1).

### 2.2. There Is a High Expression of Cytokines in Selected Areas of the Brain during Pulmonary TB

The hypothalamus, hippocampus and cerebellum are brain structures that have a critical function in various neural processes among which are motor function, cognition, learning, memory and behaviour. The expression of pro and anti-inflammatory cytokines in these brain regions was determined during the course of pulmonary TB. Interestingly, in the absence of cultivable bacteria in the brain, we found important changes in the levels of cytokine expression.

**Figure 2:**
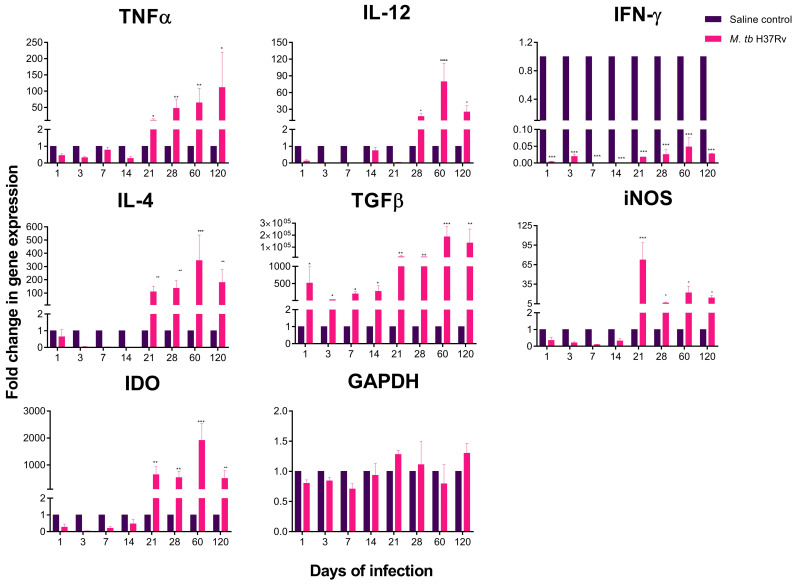
Cytokine and enzyme expression of the hypothalamus of mice intratracheal infected with *M. tb* H37Rv. In the absence of any detectable brain infection, there was a significant increase in gene expression from day 21 post-infection. Saline controls were mainly unaffected. RNA was isolated from hypothalamus homogenates and reverse-transcribed to cDNA, then analysed for changes in gene expression determined by reverse transcription-polymerase chain reaction (RT-PCR) of the indicated cytokine or enzyme. Fold-change values were normalised to expression levels of the saline controls. Data are expressed as mean ± standard error of the mean (SEM). TNFα F (1, 10) = 59.01, *p* < 0.001, two-way ANOVA; * *p* < 0.01, ** *p* = 0.005 Sidak’s multiple comparisons test (α = 0.05) (*n* = 6). IL-12 F (1, 10) = 224.3, *p* < 0.005, two-way ANOVA; * *p* < 0.01, **** *p* < 0.0001, Sidak’s multiple comparisons test (α = 0.05) (*n* = 6). IFNγ F (1, 10) = 96.9, *p* < 0.0001, two-way ANOVA; *** *p* < 0.001, Sidak’s multiple comparisons test (α = 0.05) (*n* = 6). IL-4 F (1, 10) = 84.6, *p* = 0.0089, two-way ANOVA; ** *p* < 0.001, *** *p* < 0.0001, Sidak’s multiple comparisons test (α = 0.05) (*n* = 6). TGFβ F (1, 10) = 102.3, *p* < 0.0001, two-way ANOVA; * *p* < 0.01, ** *p* < 0.001, *** *p* < 0.0001, Sidak’s multiple comparisons test (α = 0.05) (*n* = 6). iNOS F (1, 10) = 82.6, *p* < 0.001, two-way ANOVA; * *p* < 0.01, *** *p* < 0.0001, Sidak’s multiple comparisons test (α = 0.05) (*n* = 6). IDO F (1, 10) = 86.7, *p* < 0.0001, two-way ANOVA; ** *p* < 0.001, *** *p* < 0.0001, Sidak’s multiple comparisons test (α = 0.05) (*n* = 6).

The site of action of peripheral cytokines in the CNS is the hypothalamus, where a significant increase in the expression of TNFα, interleukin-12 (IL-12), induced nitric oxide synthase (iNOS) and indolamine 2,3-dioxygenase (IDO) was observed from day 21 post-infection when the peak of maximal immune protection in the lungs was observed in this murine model [10]. In contrast, the expression of IFNγ was significantly decreased in the tuberculous animals in all the studied time points in comparison with the non-infected control animals. There were also high levels of anti-inflammatory cytokines, such as transforming growth factor type-Beta (TGFβ) from day 1 post-infection and IL-4 from day 21 (Figure 2), which suggest active anti-inflammatory response mediated by cytokines.

**Figure 3:**
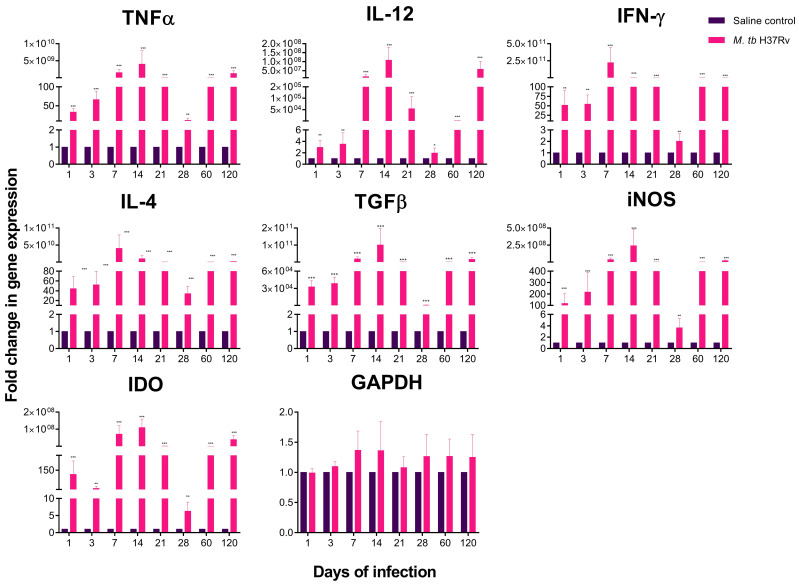
Cytokine and enzyme expression of the hippocampus of mice intratracheal infected with *M. tb* H37Rv. In the absence of any detectable brain infection, there was a significant increase in gene expression from day 1 post-infection, being the highest on days 7 and 14 post-infection. Saline controls were mainly unaffected. RNA was isolated from hippocampus homogenates and reverse-transcribed to cDNA, then analysed for changes in gene expression determined by RT-PCR of the indicated cytokine or enzyme. Fold-change values were normalised to expression levels of the saline controls. Data are expressed as mean ± SEM. TNFα F (1, 10) = 81.02, *p* < 0.0001, two-way ANOVA; ** *p* < 0.001, *** *p* < 0.0001 Sidak’s multiple comparisons test (α = 0.05) (*n* = 6). IL-12 F (1, 10) = 59.7, *p* = 0.0032, two-way ANOVA; * *p* < 0.018, ** *p* < 0.001, *** *p* < 0.0001, Sidak’s multiple comparisons test (α = 0.05) (*n* = 6). IFNγ F (1, 10) = 83.6, *p* < 0.0001, two-way ANOVA; * *p* < 0.01, ** *p* < 0.001, *** *p* < 0.0001, Sidak’s multiple comparisons test (α = 0.05) (*n* = 6). IL-4 F (1, 10) = 79.3, *p* < 0.001, two-way ANOVA; *** *p* < 0.0001, Sidak’s multiple comparisons test (α = 0.05) (*n* = 6). TGFβ F (1, 10) = 82.7, *p* < 0.0001, two-way ANOVA; *** *p* < 0.0001, Sidak’s multiple comparisons test (α = 0.05) (*n* = 6). iNOS F (1, 10) = 94.32, *p* < 0.0001, two-way ANOVA; ** *p* < 0.0016, *** *p* < 0.0001, Sidak’s multiple comparisons test (α = 0.05) (*n* = 6). IDO F (1, 10) = 89.45, *p* < 0.0001, two-way ANOVA; ** *p* < 0.001, *** *p* < 0.0001, Sidak’s multiple comparisons test (α = 0.05) (*n* = 6).

The hippocampus is particularly susceptible to neuroinflammatory damage, which produces neuronal injury and behaviour disorder. The pathogenesis of neurodegenerative diseases is strongly related to the neuroinflammation process in the hippocampus and to the development of anxiety, depression, impaired cognition and mood [22]. Our results showed that the hippocampus of TB mice exhibited a highly significant increase in the expression of the pro-inflammatory (TNFα, IFNγ, IL12) and anti-inflammatory cytokines (IL4, TGFβ), as well as the enzymes iNOS and IDO from day one post-infection (Figure 3). Interestingly, the most significant increase in cytokine expression in the hippocampus occurred on days 7, 14, and 21 post-infection, coinciding with the peak of cellular immune response and the production of type 1 cooperating lymphocytes (Th1) cytokines in the lung in this TB experimental model [10].

**Figure 4:**
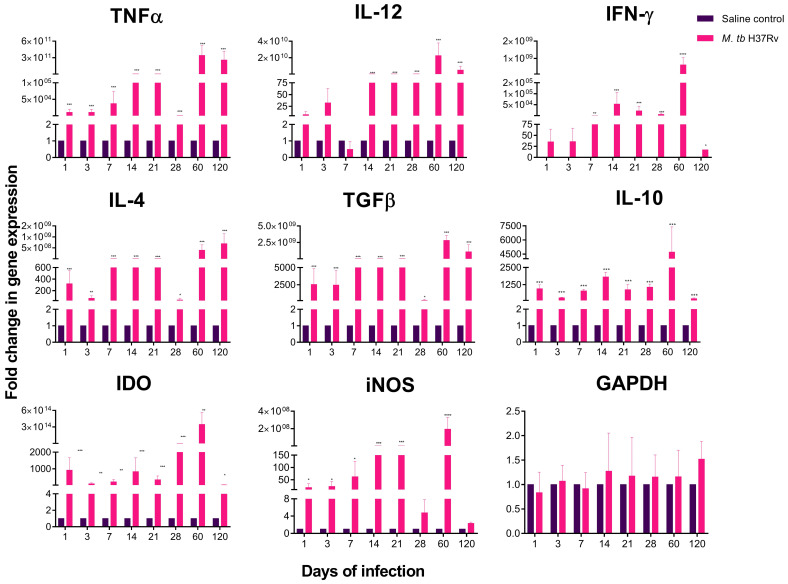
Cytokine and enzyme expression of the cerebellum of mice intratracheal infected with *M. tb* H37Rv. In the absence of any detectable brain infection, there was a significant increase in gene expression from day 1 post-infection with a peak on days 60 and 120. Saline controls were mainly unaffected. RNA was isolated from cerebellum homogenates and reverse-transcribed to cDNA, then analysed for changes in gene expression determined by RT-PCR of the indicated cytokine or enzyme. Fold-change values were normalised to expression levels of the saline controls. Data are expressed as mean ± SEM. TNFα F (1, 10) = 68.02, *p* < 0.001, two-way ANOVA; *** *p* < 0.0001 Sidak’s multiple comparisons test (α = 0.05) (*n* = 6). IL-12 F (1, 10) = 77.4, *p* = 0.0028, two-way ANOVA; *** *p* < 0.0001, Sidak’s multiple comparisons test (α = 0.05) (*n* = 6). IFNγ F (1, 10) = 48.96, *p* < 0.0001, two-way ANOVA; * *p* < 0.01, ** *p* < 0.001, *** *p* < 0.0001, Sidak’s multiple comparisons test (α = 0.05) (*n* = 6). IL-4 F (1, 10) = 85.23, *p* < 0.001, two-way ANOVA; * *p* < 0.01, ** *p* < 0.001, *** *p* < 0.0001, Sidak’s multiple comparisons test (α = 0.05) (*n* = 6). TGFβ F (1, 10) = 54.36, *p* < 0.001, two-way ANOVA; * *p* < 0.019, *** *p* < 0.0001, Sidak’s multiple comparisons test (α = 0.05) (*n* = 6). IL-10 F (1, 10) = 91.23, *p* < 0.0001, two-way ANOVA; *** *p* < 0.0001, Sidak’s multiple comparisons test (α = 0.05) (*n* = 6). iNOS F (1, 10) = 45.23, *p* < 0.0001, two-way ANOVA; * *p* < 0.01, *** *p* < 0.0001, Sidak’s multiple comparisons test (α = 0.05) (*n* = 6). IDO F (1, 10) = 76.4, *p* < 0.0001, two-way ANOVA; * *p* < 0.01, ** *p* < 0.001, *** *p* < 0.0001, Sidak’s multiple comparisons test (α = 0.05) (*n* = 6).

Neuroinflammation in the cerebellum results in mild cognitive and motor impairment [23]. Our results showed in the cerebellum a significant increase in the expression of TNFα and IFNγ from day 1 and for IL-12 from day 14 post-infection (Figure 4). The anti-inflammatory cytokines (IL-4, TGFβ) and the enzymes iNOS and IDO also showed significantly higher expression from day one post-infection. The anti-inflammatory cytokine IL-10 was up-regulated in the cerebellum of the infected animals and progressively increased from day one, raising its peak on day 60 post-infection (Figure 4).

Together, these data suggest that peripheral inflammation developed during the pulmonary infection with *M. tb* generates inflammation in the hypothalamus, hippocampus and cerebellum. The hippocampus, as we mentioned above, has an early increase in cytokine expression, when in this murine TB model, the Th1 cytokines have their highest expression at the site of infection [10]. At the same time, the hypothalamus presents a more considerable increase in the inflammatory response once the immunological response in the lung reaches its protective peak, on day 21 post-infection [10]. In the case of the cerebellum, although there was an increase in cytokines from day 1 post-infection, there was a more significant increase on days 60 and 120 post-infection. All this indicates that the hippocampus is more sensible to peripheral inflammation than the hypothalamus and the cerebellum. 

### 2.3. Pulmonary TB Affected in the Brain Components of the MAPK Pathway, Increasing the Activation of p38 and JNK and Decreasing BNDF Production

The mitogen-activated protein kinase (MAPK) cascades are intracellular signal transduction pathways that are evolutionarily conserved. These pathways respond to numerous external stimuli and regulate cellular mechanisms related to stress response and inflammation, among others. The role of MAPKs in inflammation is primarily related to the control of cytokine expression [24] and cell death. MAPK cascade comprises extracellular signal-regulated kinase (ERK) 1/2, c-Jun NH2-terminal kinase (JNK), and p38 [25]. The JNK family participates in neuroinflammation, neurodegeneration and kainate-induced excitotoxicity by regulating the expression of pro-inflammatory cytokines (TNFα, IL-2) and the decline of trophic molecules [26]. Another member of the MAPK cascades is p38; a serine kinase involved in mechanisms related to inflammatory responses, such as induced by oxidative stress and pro-inflammatory cytokines (TNFα, IL-1β) [27]. Due to the increase in pro-inflammatory cytokines and neuronal damage in the hippocampus, hypothalamus and cerebellum of tuberculous animals, we studied the JNK and p38 activation by the expression of both proteins in their phosphorylated form in the selected brain regions by Western blot on days 28, 60 and 120 post-infection. 

**Figure 5:**
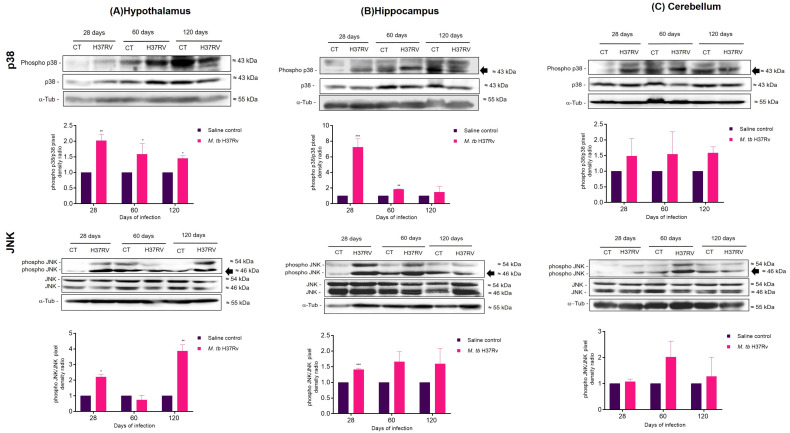
The activation pattern of MAPKs during pulmonary infection with *M. tb* at 28, 60 and 120 days post-infection in the hypothalamus (**A**), hippocampus (**B**) and cerebellum (**C**). Upper panel shows representative Western blots of activated JNK and p38. Lower panel shows the quantification of the pixel density ratio (PD ratio). There is a tendency to increase the activation of p38 and JNK in the infected mice. Data are expressed as mean ± SEM. p38 Hypothalamus 28 days *t* = 8.328, ** *p* = 0.0011, two-tail, unpaired t-test. p38 Hypothalamus 60 days *t* = 7.428, * *p* = 0.012, two-tail, unpaired *t*-test. p38 Hypothalamus 120 days *t* = 5.19, ** *p* = 0.0020, two-tail, unpaired t-test. p38 Hippocampus 28 days *t* = 6.873, *** *p* = 0.0010, two-tail, unpaired t-test. p38 Hippocampus 60 days t = 16.43, ** *p* =< 0.0001, two-tail, unpaired *t*-test. JNK Hypothalamus 28 days *t* = 8.518, * *p* = 0.022, two-tail, unpaired *t*-test. JNK Hypothalamus 120 days *t* = 9.23, ** *p* = 0.0027, two-tail, unpaired *t*-test. JNK Hippocampus 28 days *t* = 13.75, *** *p* = 0.0008, two-tail, unpaired *t*-test (*n* = 3).

Our results in tuberculous mice showed that phosphorylation of p38 and JNK increased on days 28 and 60 post-infection but did not change on day 120 in the hippocampus. These results coincide with the increase in cytokines in the hippocampus. The decrease in active p38 and JNK observed on day 120 could be related to the higher expression of anti-inflammatory cytokines. In the hypothalamus, we observed that infected animals showed an increase in active p38 at the three evaluated time points. Regarding JNK, the hypothalamus of TB animals showed an increase in the phosphorylation of this kinase on days 28 and 120 post-infection, when the highest increase in pro-inflammatory cytokines, mainly TNFα, was observed. A tendency to increase the levels of active p38 was observed in the cerebellum of the infected animals at the three evaluated times, although this was not significant. In the case of JNK, the phosphorylated form of this kinase had a higher expression on days 60 and 120, although this increase was not significant. However, it coincides with the increase in pro-inflammatory cytokines in this brain region (Figure 5).

**Figure 6:**
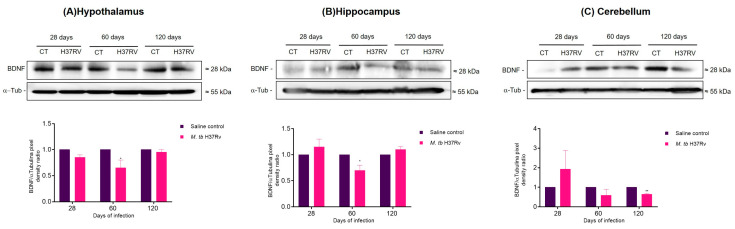
Production of BDNF in the hypothalamus (**A**), hippocampus (**B**) and cerebellum (**C**) of mice with pulmonary tuberculosis (TB). Upper panel shows a representative image of the Western blot at 28, 60 and 120 days post-infection. Lower panel shows the quantification of the PD ratio. BDNF levels decreased in the hippocampus and the hypothalamus at 60 days post-infection and in the cerebellum at 120 days post-infection. Data are expressed as mean ± SEM. Hypothalamus 60 days *t* = 3.025, * *p* = 0.0186, two-tail, unpaired t-test. Hippocampus 60 days *t* = 4.025, * *p* = 0.0276, two-tail, unpaired *t*-test. Cerebellum 120 days *t* = 9.391, ** *p* = 0.0026. (*n* = 3).

The MAPK signalling cascade regulates neurotrophic factors in the brain, mainly the brain-derived neurotrophic factor (BDNF) which regulates not only survival, development and preservation of neurons but also the survival of mature neuronal cells [28]. In addition, BDNF plays a role in learning and memory, and its decreased production is a pathogenetic factor of major depression [28]. Recently it has been demonstrated that JNK affects the levels of BDNF [29]. The results in the present work showed that pulmonary infection with *M. tb* decreased BNDF levels in the hippocampus and hypothalamus after 2 months of infection and in the cerebellum at 120 days post-infection (Figure 6). 

These results suggested that the infection with *M. tb* affected components of the MAPK pathway, increasing the activation of p38 and JNK and decreasing BNDF levels.

### 2.4. Pulmonary TB Is Related to Neuronal Injury during Early Infection and Neuronal Death in the Hippocampus and BBB Dysfunction during Progressive Late Disease

**Figure 7:**
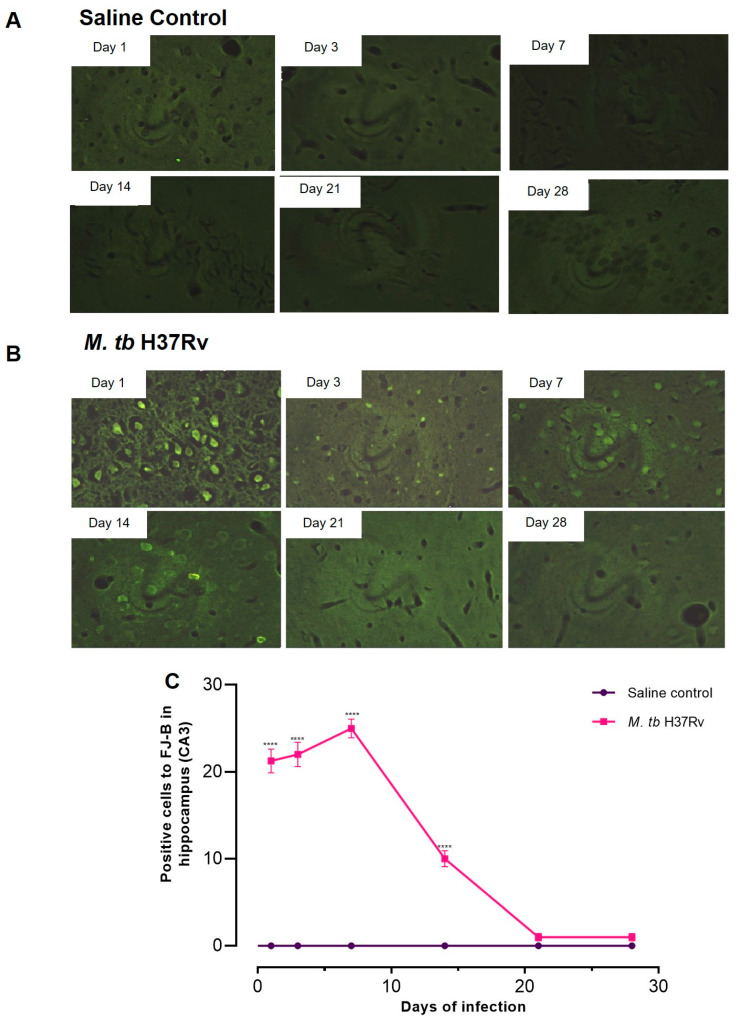
Pulmonary infection with *M. tb* induced neuronal injury in the hippocampus (CA3) at the early phase of infection. (**A**) Representative images of the Fluoro-Jade B (FJ-B) staining in CA3 in the saline control and tuberculous mice (**B**) at different time points after *M. tb* infection. (**C**) Quantification of positive cells to FJ-B in the hippocampus at the indicated days post-infection. Only infected pulmonary animals showed positive cells to FJ-B during the first and second week. Data are expressed as mean ± SEM. F (1, 6) = 2360, *p* < 0.0001, two-way ANOVA; **** *p* < 0.0001, Sidak’s multiple comparisons test (α = 0.05) (*n* = 6).

Neuroinflammation produces neuronal damage and death. Thus, we evaluated cellular injury in the brain of mice with pulmonary TB using Fluoro-Jade B (FJ-B) staining that labels the injured but not dead neurons, including cell body, dendrites and axon. Our results showed that the hippocampus, mainly the C3 region, showed injured neurons on days 1, 3, 7 and 14. In later days, no positive cells to FJ-B were seen (Figure 7). Injured neurons in the hippocampus during the early phase of infection coincide with the higher expression of inflammatory cytokines in this brain region. In the hypothalamus and cerebellum of infected animals, there were no FJ-B positive cells. 

**Figure 8:**
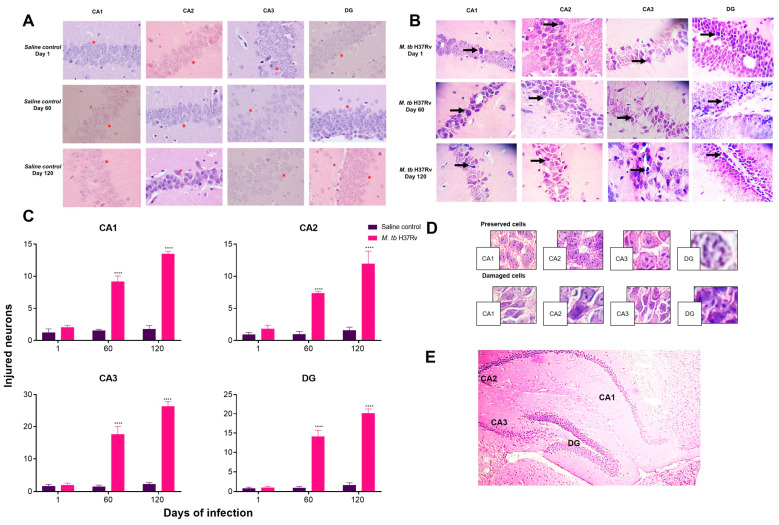
Pulmonary infection with *M. tb* induced neuronal death in the hippocampus in the late phase of infection. Representative image of the saline group (**A**) and tuberculous mice (**B**) with H&E staining (40×). The asterisks (*) in 8A show preserved hippocampal cells. Arrows indicate damaged cells. (**C**) The percentage of cells injured per field. (**D**) The criteria used to discriminate damaged cells from healthy cells. (**E**) A coronal section of the mice brain, indicating the hippocampus areas from where the fields were obtained for quantification. The infected mice presented a critical number of injured cells in the hippocampus, mostly in the CA3 region and in the DG in contrast to the saline group. Data are expressed as mean ± SEM. CA1 F (1, 10) = 218.6, *p* < 0.0001, two-way ANOVA; **** *p* < 0.0001, Sidak’s multiple comparisons test (α = 0.05) (*n* = 6). CA2 F (1, 10) = 49.01, *p* < 0.0001, two-way ANOVA; **** *p* < 0.0001, Sidak’s multiple comparisons test (α = 0.05) (*n* = 6). CA3 F (1, 10) = 268.3, *p* < 0.0001, two-way ANOVA; **** *p* < 0.0001, Sidak’s multiple comparisons test (α = 0.05) (*n* = 6). DG F (1, 10) = 106.9, *p* < 0.0001, two-way ANOVA; **** *p* < 0.0001, Sidak’s multiple comparisons test (α = 0.05) (*n* = 6).

Neuronal death was determined in regions CA1, CA2, CA3, and dentate gyrus (DG) of the hippocampus of tuberculous animals according to several histological parameters, such as basophilic pyknotic or fragmented nuclei, and acidophilic shrinking cytoplasm. The histology of hippocampus during the early phase of infection was well preserved, while during late infection on day 60, neurons with pyknotic nuclei and acidophilic cytoplasm were observed in the four regions being the highest at day 120 in the four studied areas. The hippocampus regions most affected on days 60 and 120 were CA3, and the DG (Figure 8). 

**Figure 9:**
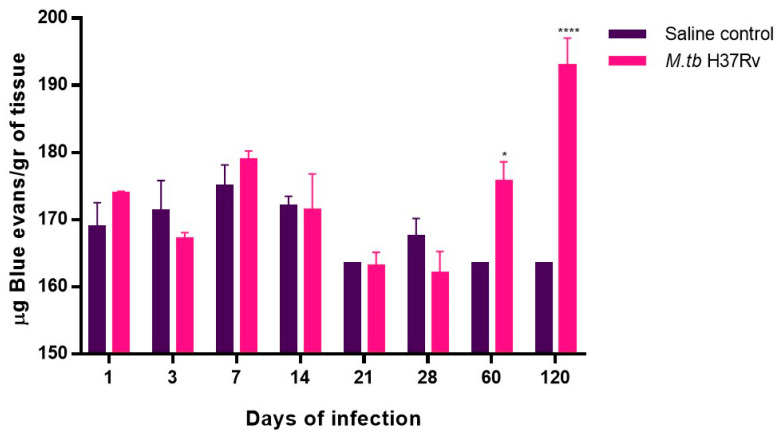
Advanced TB enhanced blood–brain barrier permeability in BABL/c mice. Simple effects analyses revealed increased extravasation of EB in the infected group at 60 and 120 days post-infection compared with the saline control. Data are expressed as mean ± SEM. F (1, 4) = 23.45, *p* = 0.0084, two-way ANOVA; * *p* < 0.01, **** *p* < 0.0001, Sidak’s multiple comparisons test (α = 0.05) (*n* = 6).

Inflammation induces BBB damage due to the increase in pro-inflammatory cytokines [30]. Thus, the permeability of the BBB was determined in tuberculous animals using the Evans Blue (EB) dye. There was a significant increase in the BBB permeability at days 60 and 120 post-infection (Figure 9). 

### 2.5. There Are Significant Changes in the Concentrations of Neurotransmitters during Pulmonary TB

In the present work, the neurotransmitters Norepinephrine (NE), Dopamine (DA), Epinephrine (EP) and 5-HT were measured in the hippocampus, hypothalamus and cerebellum of mice with pulmonary TB without cultivable bacteria in the brain.

**Figure 10:**
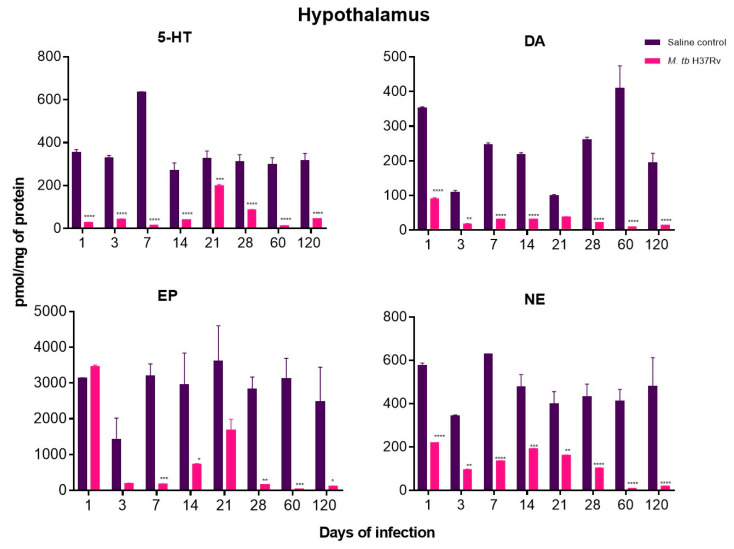
The concentration of the indicated neurotransmitter determined by HPLC in the hypothalamus of animals intratracheally infected with *M. tb*. Pulmonary TB mice showed a significant decrease in Norepinephrine (NE), Dopamine (DA), Epinephrine (EP) and 5-HT from day one of infection. Data are expressed as mean ± SEM. The following were used: 5-HT F (1, 4) = 3570, *p* < 0.0001, two-way ANOVA; *** *p* < 0.0003, **** *p* < 0.0001, Sidak’s multiple comparisons test (α = 0.05) (*n* = 6). DA F (1, 4) = 630.9, *p* < 0.0001, two-way ANOVA; ** *p* < 0.0091, **** *p* < 0.0001, Sidak’s multiple comparisons test (α = 0.05) (*n* = 6). EP F (1, 4) = 512.6, *p* < 0.0001, two-way ANOVA; * *p* < 0.0113, ** *p* < 0.0032, *** *p* < 0.0006, Sidak’s multiple comparisons test (α = 0.05) (*n* = 6). NE F (1, 4) = 443.9, *p* < 0.0001, two-way ANOVA; ** *p* < 0.0029, **** *p* < 0.0001, Sidak’s multiple comparisons test (α = 0.05) (*n* = 6).

**Figure 11:**
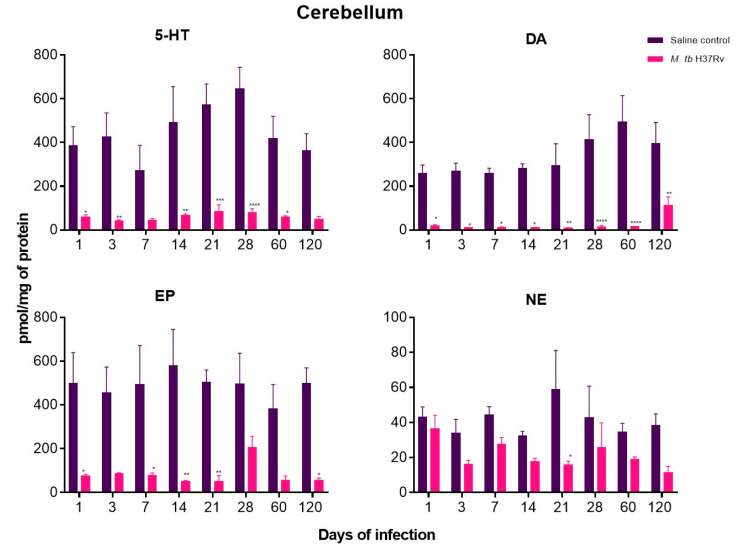
The concentration of the indicated neurotransmitter determined by HPLC in the cerebellum of animals intratracheally infected with *M. tb*. Infected mice showed a significant decrease in NE, DA, EP and 5-HT from day one of infection. Data are expressed as mean ± SEM. The following were used: 5-HT F (1, 6) = 54.28, *p* = 0.0003, two-way ANOVA; * *p* < 0.01, ** *p* < 0.001, *** *p* < 0.0001, **** *p* < 0.00001, Sidak’s multiple comparisons test (α = 0.05) (*n* = 6). DA F (1, 6) = 344.1, *p* < 0.0001, two-way ANOVA; * *p* < 0.01, ** *p* < 0.001, **** *p* < 0.0001, Sidak’s multiple comparisons test (α = 0.05) (*n* = 6). EP F (1, 6) = 215.7, *p* < 0.0001, two-way ANOVA; * *p* < 0.01, ** *p* < 0.001, Sidak’s multiple comparisons test (α = 0.05) (*n* = 6). NE F (1, 6) = 18.67, *p* = 0.0050, two-way ANOVA; * *p* < 0.01, Sidak’s multiple comparisons test (α = 0.05) (*n* = 6).

In contrast with control animals, mice with pulmonary TB showed a significant decrease in NE, DA, EP and 5-HT in the hypothalamus and cerebellum from day one of infection, when in this model there is a high production of pro-inflammatory cytokines in the lungs, such as TNF-α and IL-1-β [31]. The decrease in neurotransmitter concentrations was more pronounced at days 60 and 120 post-infection, these point times correspond in this model to the late progressive phase of the disease that is characterised by high bacillary burdens in the lungs and extensive pneumonia [10]. High production of cytokines during neuroinflammation is involved in alterations of DA synthesis and neurotransmission. Pro-inflammatory cytokines such as IFN-γ and TNF-α can up-regulate IDO expression activating kynurenine pathway that reduces serotonin levels [32]. Thus, the increase in pro-inflammatory cytokines correlates with a decrease in this monoaminergic system (Figure 10 and Figure 11). 

**Figure 12:**
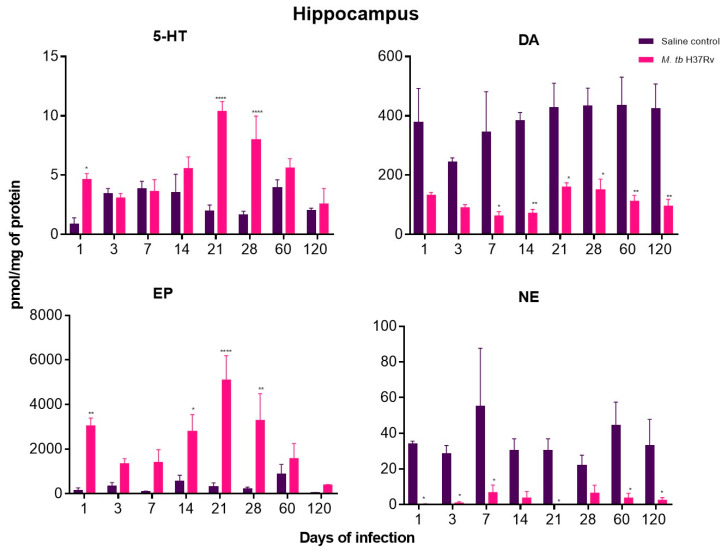
The concentration of the indicated neurotransmitter determined by HPLC in the hippocampus of animals intratracheally infected with *M. tb*. Pulmonary TB mice showed a significant increase in EP from day one of infection and a decrease in the concentration of DA and NE. Data are expressed as mean ± SEM. The following were used: 5-HT F (1, 6) = 74.16, *p* = 0.0001, two-way ANOVA; * *p* < 0.01, **** *p* < 0.0001, Sidak’s multiple comparisons test (α = 0.05) (*n* = 6). DA F (1, 6) = 60.82, *p* = 0.0002, two-way ANOVA; * *p* < 0.01, ** *p* < 0.001, Sidak’s multiple comparisons test (α = 0.05) (*n* = 6). EP F (1, 6) = 38.81, *p* = 0.0008, two-way ANOVA; * *p* < 0.01, ** *p* < 0.001, **** *p* < 0.0001, Sidak’s multiple comparisons test (α = 0.05) (*n* = 6). NE F (1, 6) = 35.01, *p* = 0.0010, two-way ANOVA; * *p* < 0.01, Sidak’s multiple comparisons test (α = 0.05) (*n* = 6).

DA and NE decreased in the hippocampus during the whole course of pulmonary TB. At the same time, EP concentrations showed a significant increase from day one of infection, raising its peak at day 21 when in this TB model there is the maximal immune protection manifested by well-formed pulmonary granulomas with high production of IFN-γ and TNF-α, as well as activation of HPA axis [10,31,33]. Interestingly, the concentration of NE was significantly lower at day 120 of infection when in this model, where there is the highest pulmonary bacillary burdens and extension of pneumonia [10]. Contrary to the hypothalamus and cerebellum, the hippocampus did not show a decrease in serotonin levels (Figure 12).

### 2.6. Pulmonary TB Is Associated with Diverse Behavioural Abnormalities 

**Figure 13:**
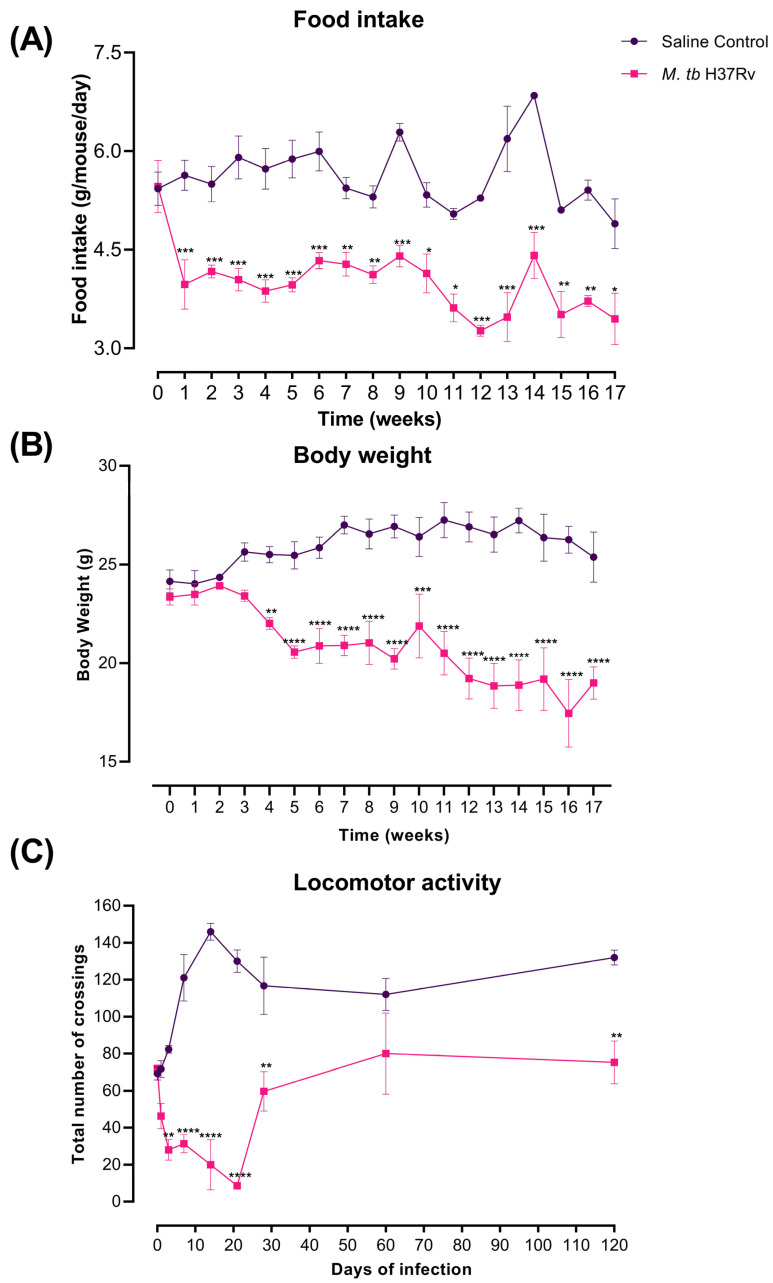
Sickness behaviour showed by TB mice. (**A**) Food intake, (**B**) body weight loss and (**C**) locomotor activity (LMA). Infected animals showed food intake decrease after one week of infection, Body weight starts decreasing after three weeks post-infection, and the locomotor activity was decreased during the whole course of the disease being most pronounced during early infection from day 1 to 21. Data are expressed as mean ± SEM. Food intake F (1, 10) = 51.86, *p* < 0.0001, two-way ANOVA; * *p* < 0.01, ** *p* < 0.001, *** *p* < 0.0005, Sidak’s multiple comparisons test (α = 0.05) (*n* = 6). Body weight loss F (1, 3) = 101.2, *p* = 0.0021, two-way ANOVA; ** *p* < 0.001, *** *p* < 0.0002, **** *p* < 0.0001, Sidak’s multiple comparisons test (α = 0.05) (*n* = 6). LMA F (1, 4) = 222.9, *p* = 0.0001, two-way ANOVA; ** *p* < 0.001, **** *p* < 0.0001, Sidak’s multiple comparisons test (α = 0.05) (*n* = 6).

The decrease in body weight, locomotor activity (LMA) and food intake are typical features of sickness behaviour. In our murine model of pulmonary TB, there was a progressive reduction in body weight after three weeks of infection. Infected mice presented a reduction in LMA during the 4 months of infection, being higher from day 1 to 21. Infected animals also showed decreased food intake after one week of infection (Figure 13).

**Figure 14:**
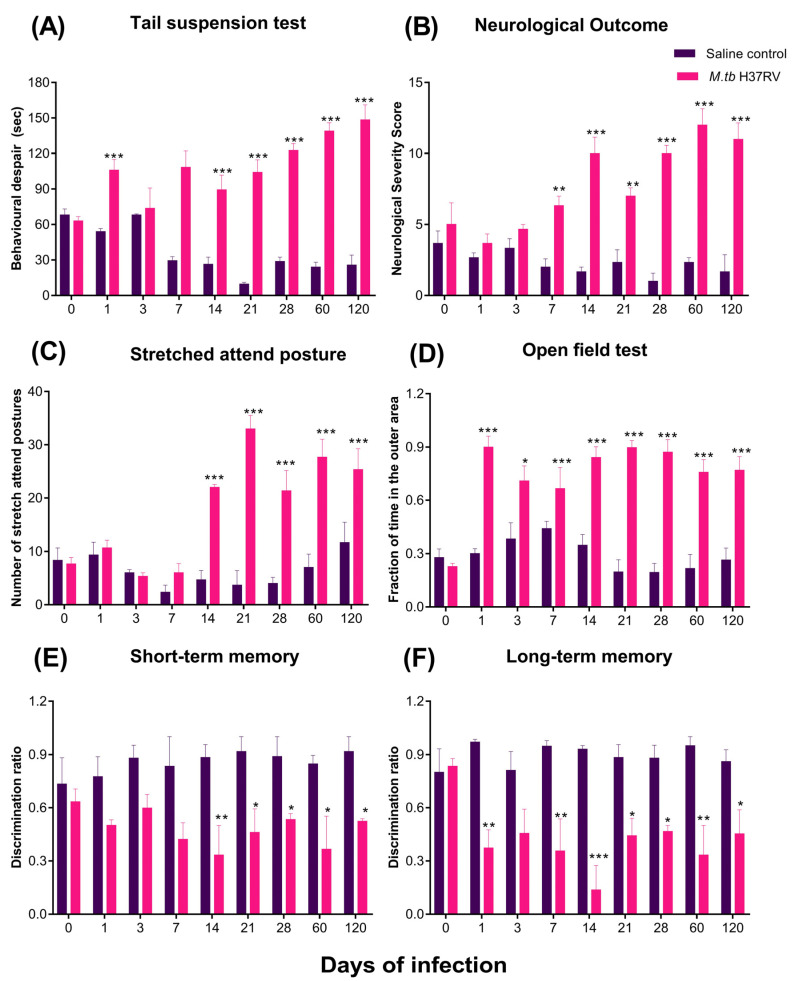
Behaviour abnormalities showed by pulmonary TB mice. (**A**) Tail suspension test (depression-like behaviour), (**B**) neurological outcome, (**C**) stretched attend posture (anxiety-like behaviour), (**D**) open field test (unconditioned fear and anxiety), (**E**) short-term and (**F**) long-term memory. Animals infected with *M. tb* in the lungs present behavioural abnormalities such as depression-like behaviour, anxiety-like behaviour, neurological damage, and short- and long-term memory damage. Data are expressed as mean ± SEM. Tail suspension test F (1, 4) = 225.2, *p* = 0.0001, two-way ANOVA; *** *p* < 0.0001, Sidak’s multiple comparisons test (α = 0.05) (*n* = 6). Neurological outcome F (1, 4) = 186.3, *p* = 0.0002, two-way ANOVA; ** *p* < 0.001, *** *p* < 0.0001, Sidak’s multiple comparisons test (α = 0.05) (*n* = 6). Stretched attend posture F (1, 4) = 166, *p* = 0.0002, two-way ANOVA; *** *p* < 0.0001, Sidak’s multiple comparisons test (α = 0.05) (*n* = 6). Open field test F (1, 6) = 326.5, *p* < 0.0001, two-way ANOVA; * *p* < 0.01, *** *p* < 0.0001, Sidak’s multiple comparisons test (α = 0.05) (*n* = 6). Short-term memory F (1, 4) = 49.05, *p* = 0.0022, two-way ANOVA; * *p* < 0.01, ** *p* < 0.001, Sidak’s multiple comparisons test (α = 0.05) (*n* = 6). Long-term memory F (1, 4) = 116, *p* = 0.0004, two-way ANOVA; * *p* < 0.01, ** *p* < 0.001, *** *p* < 0.0001, Sidak’s multiple comparisons test (α = 0.05) (*n* = 6).

Depression-like behaviour was evaluated using the tail suspension test. There was a significant increase in the time spent in behavioural despair in the group of tuberculous animals, indicative of depression-like behaviour, being the highest during late progressive pulmonary TB at day 60 and 120 of infection (Figure 14A). We also evaluated the neurological outcome (NSS). Infected animals showed higher NSS than control animals, so the pulmonary infection impaired motor activity and reflexes, from day 7 until day 120 post-infection (Figure 14B), similar to the depressive-like behaviour. 

Regarding anxiety and unconditioned fear, the stretches attend postures (SAPs), meaning anxiety-like behaviour, increased significantly from 14 days of infection and until day 120 post-infection (Figure 14C). In the open field test, we observed that animals presented anxiety and unconditioned fear from day 1 post-infection until day 120 post-infection (Figure 14D).

Finally, we evaluated the effect of the infection in memory. The results showed that pulmonary infection with *M. tb* induces damage in short-term memory from day 14 post-infection; however, damage is observed from day 1 post-infection in long-term memory (Figure 14E,F).

### 2.7. The Immune Response in the Hippocampus Is Correlated with the Presence of Behavioural Changes during Pulmonary TB

The results above prompted us to investigate whether cytokine levels in hypothalamus, hippocampus and cerebellum correlate with the behavioural changes observed in infected TB animals. Correlation analysis between the behavioural scores in each test and cytokine levels revealed a strong association of sickness behaviour (LMA) with TNF-α, IL-12, IL-4 and TGFβ in the cerebellum and with TNFα, IL-12, IFNγ and IL4 in the hypothalamus. Depression (TST) is associated with the presence of TNF-α, IL-12 and IL-4 in the cerebellum and with TNFα in the hypothalamus. However, the high expression of cytokines in the hippocampus correlated well with most of the behavioural abnormalities. Overexpression of TNFα and IL-12 in the hippocampus had a strong association with damage in short- and long-term memory and with depression, anxiety (SAP) and unconditioned fear (OFT). Interestingly, the levels of IFNγ in this brain section are strongly related to anxiety and unconditioned fear and TGFβ and iNOS with the damage in the long-term memory (Table 1). 

## 3. Discussion

TB is an infectious/contagious disease that affects mostly the lungs producing alterations in the immune system, and characteristically induces chronic inflammation. In recent years, it has been noticed that TB has a strong relation to depression and anxiety. However, the mechanisms responsible for this comorbidity are not well understood. Current advances in neuroscience research have shown a complex network of communication pathways between the CNS and the immune system [34]. Specifically, there is a relationship of systemic inflammation with the development of neuroinflammation, neurodegeneration, neurodegenerative diseases and psychiatric diseases such as depression and anxiety [35]. Thereby, we decided to evaluate the effect of pulmonary infection with *M. tb* on the immune response in the CNS and its relationship with behavioural changes.

Peripheral pro-inflammatory cytokines can signal the brain by active transport through the BBB, the choroid plexus, or by afferent nerves such as the vagus nerve [36,37,38]. These peripheral inflammatory signals stimulate brain cells such as neurons, microglia and astrocytes to respond with the same pro-inflammatory cytokines production [39]. To corroborate this, we evaluated the incidence of brain pro-inflammatory cytokines during pulmonary infection with *M. tb*. We observed a significantly higher synthesis of cytokines in the hypothalamus, hippocampus, and cerebellum that followed the kinetics of lung inflammation during experimental pulmonary TB. On the other hand, it is well-documented that *M. tb* H37Rv cannot access the brain due to the protection of the BBB [21]. Thus, the *M. tb*-induced neuroinflammation is probably an indirect effect of lung inflammation. Similar results were found in a study that used multiple low doses of LPS administration; it found that systemic inflammation generated by LPS induces a significant rise in the brain levels of TNFα, IL-6, IL-10 and TGFβ [40]. Similar results were found in murine models infected with *Plasmodium falciparum* [41] or coinfected with *Toxocara canis* and *Toxoplasma gondii* [42,43] that induced an increase in TNFα, IFNγ, IL10 and IL4 brain production following infection with these parasites. Obesity caused by a high-fat diet (HFD) has also been related to the release of pro-inflammatory cytokines in the hypothalamus and hippocampus, leading to leptin and insulin insensitivity and cognitive impairment [44]. As we can see, peripheral inflammation is critical in the development of CNS inflammation. Thus, we can say from our results that even in the absence of bacteria in the brain, the lung immune response produces a neuroinflammation state that may be related to neuronal damage.

Once we evaluated the immune response in the hypothalamus, hippocampus and cerebellum of TB mice, we wanted to know the molecular mechanism related to the increase in cytokines in the brain. MAPKs are proteins that have a crucial function in regulating the production of inflammatory mediators [25]. Moreover, the activity of coordinate MAPK in microglia and astrocytes has a regulatory role in the synthesis of inflammatory cytokines mediators. The JNK and p38 pathways regulate the mRNA stability of cyclooxygenase 2 (COX-2), TNF-α, IL-3, IL-6, IL-8, monocyte chemoattractant protein-1 (MCP-1α), granulocyte-macrophage colony-stimulating factor (GM-CSF), vascular endothelial growth factor (VEGF), urokinase-type plasminogen activator (uPA), and iNOS genes [45] and contributes to microglia-induced neuronal cell death [46,47]. Thus, we evaluated the activation of these proteins during pulmonary infection with *M. tb* in the same regions that we evaluated the immune response. Our results showed a significant increase in p38 and JNK in hippocampus, hypothalamus and cerebellum during late progressive pulmonary TB. Similar results have been observed in obesity and glucose intolerance induced by an HFD where there are Alzheimer’s disease-like pathological changes in the brain with an increase in MAPK signalling [48]. We also evaluated the BDNF levels in the murine model of pulmonary TB. It is well known that in underneath neuroinflammatory conditions, BDNF levels are reduced secondary to the M1 polarisation of microglia and pro-inflammatory astrocyte secondary to NFκB activation [48]. In our murine model of TB, we observed a decrease in BDNF after 60 and 120 days of infection in hypothalamus, hippocampus and cerebellum. In fact, the pro-inflammatory microglial cell is related to low BDNF brain levels in an LPS-induced depression-like model [49,50].

On the other hand, a genomic-wide associations study identified the BDNF gene as a candidate with genetic susceptibility to TB in wild boar [51]. Data suggest that BDNF exerts anti-inflammatory effects, by suppressing the production of pro-inflammatory mediators via the inhibition of NF-κB and MAPK signalling pathways in LPS-stimulated BV-2 cells [52]. These results suggest that the decrease in the BDNF levels observed in this work could be associated with the p38 activation in the hippocampus and hypothalamus, suggesting a crucial role of BDNF in the inflammatory damage induced by *M. tb*. 

Since chronic systemic inflammation compromises BBB integrity, we next evaluated whether pulmonary TB infection had any effect on the BBB. Our results showed that after two months of infection, the permeability of BBB increased. Similar results have been found with the administration of LPS, where chronic systemic inflammation by itself affects BBB integrity [40,53]. Furthermore, metabolic syndrome and the resulting insulin and leptin resistance and hyperglycaemia have pro-inflammatory effects with profound consequences on the BBB [54]. Thus, peripheral inflammation has a relationship with the permeability of the BBB increase, which coincides with our results.

Given that activation of peripheral inflammatory responses have been associated with mood and anxiety disorders and that peripheral immune activation can induce CNS inflammation, there has been interest in the influence of cytokines and their signalling pathways on neurotransmitter systems related to depression and anxiety including 5-HT, NE, DA and glutamate [55]. Thereby, we evaluated the production of NT in the hippocampus, hypothalamus and cerebellum of TB mice. We observed a significant decrease in the concentration of 5-HT, as well as DA, NE and EP in the hypothalamus, hippocampus and cerebellum of infected animals, which was more accentuated during the late progressive phase of TB in co-existence with extensive chronic pulmonary inflammation. Similarly, peripherally administered IFN-α is capable of accessing the brain in humans and is associated with an inflammatory response in the CNS as reflected by elevations in cerebrospinal fluid (CSF) IL-6, MCP-1 and with decreases in the 5-HT metabolite, 5-HIAA, which, in turn, were correlated with depression [56].

Inflammatory cytokines can impact the synthesis of monoamine neurotransmitters by two major pathways. First, inflammatory cytokines and their signalling pathways can activate IDO [57]. IDO converts tryptophan, the primary amino acid of 5-HT, into kynurenine, thus potentially depleting the availability of 5-HT in the brain. Activation of IDO in the brain has been shown to have an important role in the development of depressive-like behaviour in mice by the administration of LPS [58] and infection with the mycobacterium, BCG [59]. This coincides with our results. We observed a significant increase in IDO expression in TB mice with the presence of depressive-like behaviour. Additionally, inflammatory cytokines have been shown to raise the expression and function of the transporters for 5-HT, NE and DA by the activity of p38 MAPK [60,61]. As we mentioned previously, infected animals increased p38 levels in the brain regions studied. Thus, it could be possible that also the decrease in neurotransmitter is related to the activation of the MAPK signalling in the pulmonary TB mice.

Summarising, our results showed that in the absence of cultivable bacteria in the brain, *M. tb* induces neuroinflammation, neuronal damage and behavioural abnormalities during pulmonary infection, which is manifested by high production of different cytokines, which disturbs the production of neurotransmitters and induces oxidative damage, with activation of p38 and JNK and a decrease in BNDF production. As a consequence, neuronal injury and death happened in the brain, and the permeability of the BBB increased. All these changes induced behavioural alterations and neuropsychiatric symptoms such as depression and anxiety. However, more research is needed in order to understand the related mechanisms in the induction of neuroinflammation during infection with *M. tb* that will let us generate new strategies for the treatment of TB patients with depression or anxiety.

## 4. Materials and Methods

### 4.1. Reagents and Antibodies

The Middlebrook 7H9 and 7H10 media and the OADC (oleic acid, albumin, dextrose and catalase) were obtained from Becton-Dickinson (Detroit MI, USA). The Rneasy^®^ Mini Kit for RNA extraction, the Omniscript^®^ Reverse Transcription Kit for obtaining complementary DNA and the QuantiTectTM SYBR^®^ for RT-PCR were purchased from Qiagen (Germantown, MD, USA). The primers of the analysed genes and pierce BCA Protein Assay kit for protein quantification were gotten from InvitrogenTM Thermo Fisher Scientific (Waltham, MA, USA). The standards for DA, 5-HT, EP, and NE, as well as ascorbic acid, L-cysteine, bovine serum albumin (BSA), phenylmethylsulfonyl fluoride (PMSF) and α-tubulin antibody (T9026), were obtained from Sigma Chemical Co. (St. Louis, MO, USA). HPLC grade acetonitrile, perchloric acid, and EDTA were purchased from JT Baker (Mexico City, Mexico). Primary antibodies against phospho-JNK (4671), JNK (9252), phospho-p38 (9215) and p38 (9212), were purchased from Cell Signaling Technology (Danvers, MA, USA). Primary antibodies against BDNF (ab72439) were obtained from Abcam Inc. (Cambridge, MA, USA). Secondary antibodies against rabbit (711-035-152) and mouse (715-035-150) were purchased from Enzo Life Sciences (Farmingdale, NY, USA). Fluoro-Jade B was purchased from Millipore (Bedford, MA, USA). All the other reagents were obtained from known commercial sources.

### 4.2. Animals

A total of 560 adult male BALB/c mice, eight weeks old, were obtained from the animal house facility of the National Institute of Medical Science and Nutrition Salvador Zubiran (INCMNSZ), Mexico. Mice were group-housed (*n* = 5/cage) and randomly divided into two groups: control (CT, *n* = 256) and infected (H37Rv, *n* = 304). All the animals were kept in an accredited animal holding facility maintained at a controlled temperature (23 ± 1 °C) and humidity (50 ± 20%) under a 12:12 h light: dark cycle (lights on at 07:00 h). Food and water were provided ad libitum. All the animal experiments were performed according to the guidelines of the ARRIVE and Mexican Constitution law NOM 062–Z00-1999, and approval by the Ethical Committee for Experimentation in Animals of the INCMNSZ (Comite Interno para el Cuidado y Uso de los Animales de Laboratorio, 14 December 2016) in Mexico protocol number: PAT-1865-16/19-1.

### 4.3. The Experimental Model of Pulmonary TB

The murine model of progressive pulmonary TB was described previously [10]. Briefly, the reference *M. tb* strain H37Rv was cultured in 7H9 medium with OADC enrichment. Mid-log-phase cultures were used for all experiments. *M. tb* strains were counted and stored at −80 °C until use. Bacterial aliquots were thawed and pulse-sonicated to remove clumps. After mice infection, the remnant of the bacterial inoculum was plated to confirm the number and viability of CFU administered to the animals. Male BALB/c mice, 8 weeks of age, were anaesthetised in a gas chamber using 0.1 mL of sevoflurane per mouse. A blunt stainless-steel cannula with a small ball in its terminal end was inserted through the mouth and directed to the trachea, proper intratracheal placement of the cannula was verified by palpation of the small ball from the cannula rubbing the tracheal rings. Mice were infected through intratracheal instillation with 2.5 × 10^5^ live bacilli.

Mice were maintained in a vertical position until spontaneous recovery. A total of 304 infected mice were maintained in groups of five in cages fitted with micro-isolators in a *p*-3 biosecurity level facility.

### 4.4. Experimental Design

**Figure 15:**
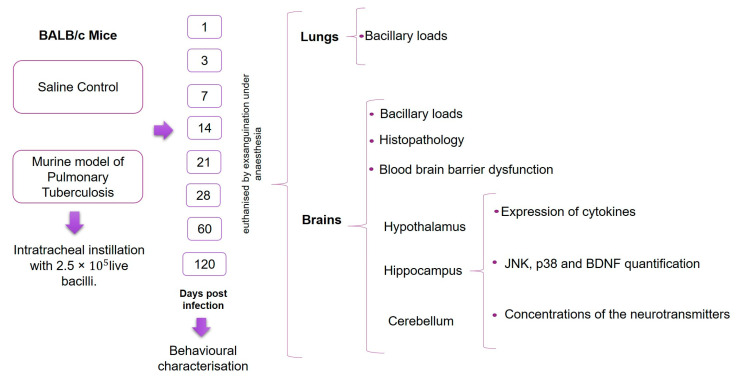
Study design workflow. BALB/c mice, 8 weeks of age, were infected with 2.5 × 10^5^ live bacilli or received saline solution (control). At days 1, 3, 7, 14, 21, 28, 60 and 120 post-infection, different behavioural tests were performed. After the behavioural tests, animals were euthanised, and the brain and lungs were collected for the determination of bacillary loads. The hypothalamus, hippocampus and cerebellum were used to determinate the cytokines gene expression, JNK, p38 and BDNF quantification and the determination of neurotransmitters levels. In brains, histological damage and the permeability of the blood–brain barrier were also measured. For each of the measurements, except for the Western Blot, two independent experiments were performed with *n* = 3 each. The samples for each experimental age group were run separately.

We analysed the effects of pulmonary TB in the CNS inflammation and its relationship with behavioural changes. Following infection, mice were euthanised by exsanguination under anaesthesia at days 1, 3, 7, 14, 21, 28, 60 and 120 post-infection; lungs and brain were collected immediately to determine bacillary loads by CFU counts. The selected areas of the brain (hypothalamus, hippocampus, cerebellum) were immediately dissected by cutting with a razor blade, according to The Mouse Brain in Stereotaxic Coordinates [62]. Briefly, the hippocampus was obtained underneath the frontal cortex, the cerebellum was identified as between the brainstem and the lateral recess of the 4th ventricle, and the hypothalamus was obtained as the area lateral and medial to the fornix. Immediately after the dissection, the sample was frozen by immersion in liquid nitrogen and used to quantify cytokines gene expression by RT–PCR, the concentrations of the neurotransmitters by HPLC, the study of the activation of MAPK signalling by Western blot assays and the histopathological alterations. A total of 256 non-infected mice (CT group) received only the vehicle (saline solution) by intratracheal route following the same procedure and were used as controls. Different behavioural tests were performed during pulmonary TB. These tests included the study of sickness behaviour (LMA, food intake and weight loss), anxiety-like behaviour, NSS, short and long-term memory and depression-like behaviour. Animals were monitored daily and were humanely euthanised under pentobarbital anaesthesia if respiratory insufficiency, accentuated cachexia, or total immobilisation was noted. The experimental protocol of the current study is represented in Figure 15. Two independent experiments were performed.

### 4.5. Determination of Colony-Forming Units (CFU) in Infected Lungs and Brain

The right lungs and right hemisphere of the brains of six mice at each time point of two independent experiments were used for bacterial colony counting. Lungs and brains were homogenised with a FastPrep homogeniser from MP Biomedicals (Irvine, CA, USA) in sterile tubes containing 1 mL of isotonic saline solution. Four dilutions of each homogenate were spread onto duplicate plates containing Bacto Middlebrook 7H10 agar, enriched with OADC. Incubation time and CFU counting were at 21 days of plating [21].

### 4.6. Expression of Cytokines by RT-PCR

Hippocampus, hypothalamus and cerebellums from six CT and infected animals at each time point were used to isolate mRNA using the RNeasy Mini Kit, according to recommendations of the manufacturer. Quality and quantity of RNA were evaluated through spectrophotometry (260/280) and on agarose gels. Reverse transcription of the mRNA was performed using 100 μg RNA, oligo dT and the Omniscript kit. Real-time PCR was performed using the 7500 RT-PCR system form Applied Biosystems (Massachusetts, USA) and Quantitec SYBR Green Mastermix kit from Qiagen (Germantown, USA). Standard curves of quantified and diluted PCR product, as well as negative controls, were included in each PCR run. Specific primers for genes encoding glyceraldehyde-3-phosphate dehydrogenase (GAPDH) as housekeeping gene and for TNF-α, IFN-γ, interleukin (IL) 12, IL-10, IL-4, TGF-β, iNOS and IDO were designed using the program Primer Express from Applied Biosystems (Massachusetts, USA). The gene expression of these cytokines was determined as previously described [21]. Cycling conditions used were: initial denaturation at 95 °C for 15 min, followed by 40 cycles at 95 °C for 20 s, 60 °C for 20 s, and 72 °C for 34 s. Quantities of the specific mRNA in the sample were measured according to the corresponding gene-specific standard. Each sample was tested in duplicate. Data were presented as the fold change in gene expression compared to the control group.

### 4.7. Study of the Activation of MAPK Signalling by Western Blot Assays

In order to study if the MAPK are involved in the cellular damage, the expressions of JNK and activated p38 (phosphorylated) and non-activated were determined by Western blot, as well as the levels of BDNF using the same technique. The hippocampus, hypothalamus and cerebellum were dissected quickly and homogenised in 500 μL of lysis buffer pH 7.9 (containing 20 mM Tris HCl, 30 mM NaCl, 0.5 mM sucrose, 1 μg/μL leupeptin, 1 μg/μL aprotinin, 1 μg/μL pepstatin, 1 μg/μL PMSF and 1 μg/μL phosphatase inhibiting cocktail) and centrifuged at 20,800× *g* for 30 min at 4° C. The supernatants were used to determine the protein quantity using Lowry’s technique and the Western blot assay. Briefly, 50 µg of the total lysate of protein was loaded and separated in 10% or 12% SDS polyacrylamide gel electrophoresis and transferred to polyvinylidene fluoride (PVDF) membranes (Millipore, Bedford, MA, USA). Membranes were blocked using 5% BSA for 2 h at room temperature with slight agitation. Blots were then incubated with anti-phospho-JNK (1:1000) at room temperature for two hours, anti-JNK (1:1000), anti-phospho-p38 (1:1000), anti-p38 (1:1000), anti-BDNF (1:1000), or anti-β-tubulin (1:8000) at 4 °C overnight. Membranes were washed three times (10 min) with TBS plus 0.1% Tween (TBS-T). A horseradish peroxidase-conjugated secondary polyclonal antibody anti-rabbit (1:10,000) and anti-mouse (1: 10,000) were then added for 2 h and after extensive washing. Bands were detected using the enhanced chemiluminescence of detection system (ECL, Amersham Pharmacia Biotech, USA). The membranes were washed with stripping solution (containing 0.2 M glycine, 0.1% SDS and 1% Tween 20, pH 2.2) for the detection of two or more proteins. The chemiluminescence imaging system Fusion Solo S (Eberhardzell, Biberach, Germany) was used. Area values were obtained from the relationship between the pixel densities (PDs) of each band. Area values of each group were standardised to the area value of the control group (value = 1). Data are expressed as the PD ratio using ImageJ software.

### 4.8. Neuronal Damage in the Hippocampus Determined by FJ-B Staining

FJ-B staining is a useful technique to determine damaged neurons that are still alive (degenerating neurons), and it was carried out according to the published protocol [63]. Briefly, tissue sections, 8 µm thick, were deparaffinised and immersed in a solution containing 1% NaOH in 80% ethanol for 5 min, followed by 2 min in 70% ethanol and 2 min in distilled water. Afterwards, the sections were immersed in a 0.06% KMnO4 solution for 10 min until moving and then cleaned in distilled water for 2 min. Tissue sections were stained with 0.0004% FJ-B solution for 20 min and washed three times with distilled water. The slides were dried in an oven at 50 °C for 15 min and cleared with xylene before coverslipping. Two random fields were selected in each mouse from the different experimental groups. Results are expressed as the number of FJ-B positive cells per field.

### 4.9. Preparation of Brain Tissue for Histological Analysis

Four mice per selected time point were anaesthetised with sodium pentobarbital (100 mg/kg, i.p.) and immediately perfused transcardially with isotonic saline solution, followed by cold 4% paraformaldehyde solution diluted with Sorensen’s phosphate buffer (0.133 M, pH 7.2). Brains were removed and postfixed in 4% paraformaldehyde for 24 h and embedded in paraffin. Coronal sections that were 4μm thick mounted on glass slides, deparaffinised, and stained with haematoxylin and eosin. For quantification of tissue damage, five fields of regions CA1, CA2, CA3, and DG of the hippocampus were analysed under a light microscope Q-win Leica 500 to estimate the number of neurons showing morphological changes indicative of cell death (cell shrinking, condensed hyperchromatic nucleus, basophilic cytoplasmic material disappearance, or cytoplasmic vacuoles). The results are presented as a percentage of death neurons in hippocampal regions.

### 4.10. Assessment of BBB Dysfunction Using Evans Blue Staining

We analysed the BBB dysfunction according to a previously published protocol [64]. Briefly, mice were anaesthetised with sodium pentobarbital (100 mg/kg, i.p.) and injected with Evans blue (EB) (2%, 2 mL/kg) in the caudal vein 3 h before perfusion. Mice were perfused transcardially with isotonic saline solution and directly beheaded, stripping brain tissue on ice. Each brain was weighed and then homogenised with a FastPrep homogeniser (MP Biomedicals) in 0.25 mL of 100% TCA and 0.75 mL of PBS solution. Samples were cooled overnight at 4 °C and then centrifuged for 30 min at 1000× *g* at 4 °C. The EB in the supernatants of 100 μL of each sample was then measured at 620 nm using a 96-well plate reader. All measurements were within the range of detection established by a standard curve. The dye concentration was considered as the ratio of absorbance relative to the amount of tissue.

### 4.11. Neurotransmitter Quantification by HPLC

The neurotransmitters’ quantification was previously described [17]. Briefly, the hippocampus, cerebellum and the hypothalamus of six CT and infected animals were homogenised using 400 µL of a solution containing 5% ascorbic acid, 200 mM sodium phosphate, 2.5 mM L-cysteine, and 2.5mM EDTA. Tissues were kept frozen to avoid enzymatic breakdown, until they were homogenised in perchloric acid (PCA), which inactivates enzyme activities. Proteins were precipitated by the addition of 100 µL of 0.4 M PCA at 4 °C, followed by incubation at 20 °C for 20 min. Supernatants containing NE, EP, DA, and 5-HT were collected after centrifugation at 16,128 g for 10 min (4 °C). NE, EP, DA, and 5-HT concentrations were determined by reversed-phase HPLC (RP-HPLC) in a system integrated by two 515 pumps (Waters™), degasser AF (Waters™), 717 autosampler (Waters™), and an X-LC™3120FP fluorescence detector (Jasco, Inc). Instruments were controlled by Millennium 32 software (Waters™). Chromatographic runs were performed using a Jupiter C18 column (300Å, five μ, 4.6 × 250mm, Phenomenex^®^) at 30 °C. The column was equilibrated with the mobile phase A (MPA) containing 0.1% trifluoroacetic acid. Mobile phase B (MPB) containing 0.1% trifluoroacetic acid in acetonitrile was used to perform a linear gradient until reaching 20% MPB, from min 5 to min 15. Then, 20% MPB was maintained until min 20; the flow rate was 0.8 mL/min. The fluorescence detector was set at gain 100, attenuation 32, response 20 s, and 280 nm and 315 nm for excitation and emission, respectively. The sample injection volume was 50 μL. The concentration of each neurotransmitter was obtained by mg of protein in each sample. The concentration of total protein was obtained with the Pierce BCA protein assay kit. The results are shown as pmol/mg of protein. The limit of detection (LOD) for norepinephrine, epinephrine, and dopamine was 2 picomoles, whereas for serotonin it was 1 picomole. The limit of quantification (LOQ) for all analytes was 5 picomoles considering a coefficient R^2^ = 0.98 for all points of the calibration curve.

### 4.12. Behaviour Tests

In order to avoid potential habituation, groups of mice were tested only once at the mentioned time points post-infection in control and infected mice. Animals were habituated to the test environment 24 h before it was made. All behavioural trials were performed during the first 4 h of the dark phase of the light cycle.

The effect of *M. tb* lung infection on LMA was evaluated in an open field. Mice were individually positioned into a clean, novel cage of 30 cm × 30 cm similar to the household pen, but devoid of bedding or litter allowing the mouse to move between compartments freely. The box was divided into sixteen quadrants, and each animal was video recorded for 10 min, LMA was measured by counting the number of crossings between quadrants. Data are represented as the total number of crosses.

In order to estimate sickness behaviour, we evaluated food intake and weight loss. Twice a week the amount of food given to mice was weighed to determine food intake, and the total ingesting of food by mice was calculated as is indicated in the next formulation where *n* is the number of mice per cage. Data are expressed as g/mouse/day.

$$Food\;intake=\frac{\left[\frac{Initial\;food\;\left(g\right)-final\;food\left(g\right)}{days\;}\right]}{n}$$

The weight loss of the animals infected with *M. tb* was estimated from day one post-infection until day 120. Each week the animals were weighted, and their loss weight recorded. Data are represented as g of body weight.

We evaluated depression-like-behaviour with the tail suspension test [65]. In this test, the animal’s immobility is named “behavioural despair”, and it is interpreted as if the mice had given hope of escaping from a stressful situation or had stopped struggling in an inescapable situation [66], and thus has been used as a model of depression [67]. For this test, animals were suspended from the tail 6 min in a tripod 30 cm height, and their activity was recorded, focusing on the time the mice spent in behavioural despair. The time that the animal presented behavioural despair for 6 min was recorded.

The anxiety-like behaviour was evaluated studying the SAP [68]. A forward elongation of the body exhibited when the animal is either standing still or moving slowly forward characterises SAP and is helpful to assess anxiety behaviour in rodents. During exploratory anxiety–conflict situations, SAP can be used as a valid measure of anxiety as anxiolytic drugs have successfully reduced SAP [69]. In this test, we used a large circular platform raised at 35–40 cm from the floor, and a small circular awning fastened by the central pillar was placed at 4 cm. Each mouse was located in the covered area of the platform and filmed for 5 min. The number of stretched attend postures was counted as a measure of anxiety-like behaviour.

Furthermore, we assessed unconditioned fear and anxiety with the open field test, which examines the innate fear of being in the central open area versus the tendency to explore new environments. When animals are anxious, they prefer to stay close to the walls [67]. For this test, we used the same open field as in the LMA test, but in this case, we recorded video from the top in a 5 min session and evaluated the time spent in the outer area. Data are presented as the fraction of time the mice spent in the outer area.

The motor function and reflexes of the infected mice were evaluated using a neurological severity score (NSS) [70]. They were valued regarding absent (0) or present (1), except for the hypomobility, motor impairment and balance that is rated as weak (1), moderate (2) or strong (3). The maximum rating was 31 (indicating neurological damage); usual was from 3 to 6 (Supplemental Table S1).

**Figure 16:**
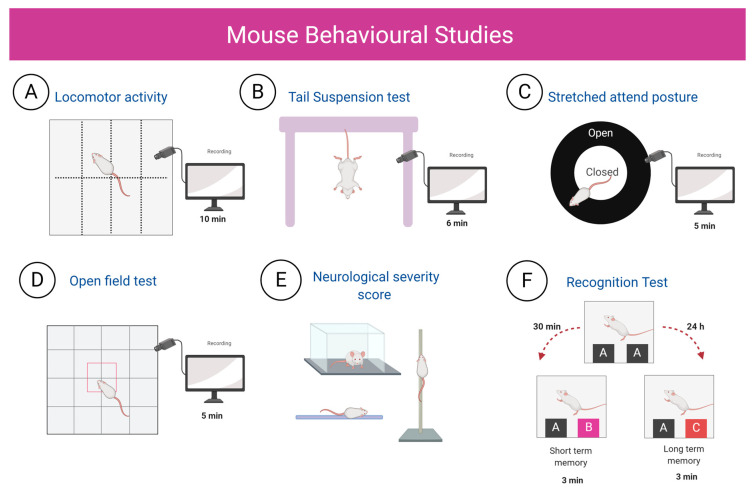
Schematic representation of the behavioural tests performed. Groups of mice of both experimental groups were tested only once, and the studies were performed during the first 4 h of the dark phase of the light cycle. (**A**) Locomotor activity. To assess LMA, the animals were placed in a box divided into 16 quadrants, and the number of crosses between quadrants were counted in 10 min. (**B**) Tail suspension test. In this test, the animals were suspended from the tail ventrally for 6 min, and the time that the animal presented behavioural despair was recorded. (**C**) Stretched attend posture. For this test, each mouse was located in the covered area of the platform and filmed for 5 min. The number of stretched attend postures was counted for 5 min. (**D**) Open field test, we videotaped from the top in a box divided into 16 quadrants by 5 min and evaluated the time spent in the outer area. (**E)** Neurological severity score. (**F**) Recognition test. For this test, the animals were exposed to two objects A, and 30 min after, one of these was changed for object B, whereby short-term memory was evaluated; for long-term memory, the same procedure was carried out, but after 24 h, object C was presented to the animals. (Created with BioRender.com.)

Memory and learning after pulmonary infection with *M. tb* were assessed with the Object Recognition Test [71]. With this test, we evaluated short-term and long-term memory. In a first habituation phase, we placed the animal in the open field without any object for 10 min to become familiar with the environment. At 24 h, two identical objects (objects A) were placed in different positions, and the animal was left inside the box for 3 min. In the next phase, 30 min later short-term memory was measured. For this, we positioned object A (familiar object) and placed a new object in the other position (object B); the interactions with both objects (the animal sniffs or touches the object with the front legs) were counted for 3 min. After 24 h, long-term memory was measured, for which object B was changed to a novel object (C), and the same procedure was followed. The results are presented as the discrimination ratio, which is the difference in interactions expressed as a proportion of the total interactions with the two objects in both tasks. The mice behavioural studies of the current study are represented in Figure 16.

### 4.13. Statistical Analysis

Data are expressed as the mean ± standard error of the mean (SEM) of two independent experiments. All data collection was randomised. All data were tested with the Shapiro–Wilk normality test and were normally distributed. Statistical significance was assessed using unpaired t-tests or two-way ANOVA, as specified in the related text. Cytokine expression by RT-PCR, BBB dysfunction, FJ-B, histological damage, behavioural assays, loss of body weight, food intake and neurotransmitter levels were analysed by two-way ANOVA followed by Sidak’s multiple comparison tests (comparison of each group against the control). Unpaired t-tests analysed Western blot assays. The correlation analysis between the behavioural score in each test and cytokine levels was made using Pearson product correlations. The media of each expression of cytokine at each time of infection was correlated with the media of each behaviour score at the same time. Statistical significance was set at *p* < 0.05 for all experiments. All main statistical results are presented in the main text, and specific statistical results are provided in Supplemental Table S2. Statistical analyses were performed in GraphPad Prism (v7) (GraphPad, San Diego, CA, USA).

## 5. Conclusions

**Figure 17:**
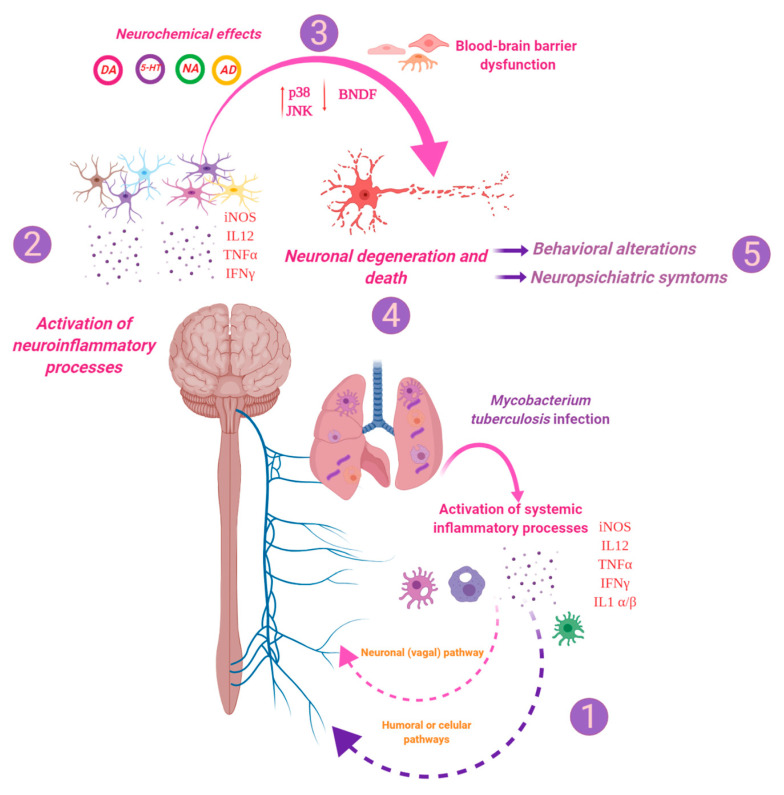
*M. tb* induces neuroinflammation, neuronal damage and behavioural abnormalities during pulmonary infection. TB is an infectious disease that mainly affects the lungs, although it can spread to other organs and cause extrapulmonary TB. However, most of the time, the host’s immune system efficiently controls the infection. Although, when there is some failure in the immune system, the infection becomes active. (1) Intense inflammation due to the immune response against mycobacteria in the lungs induces neuroinflammation by humoral and neuronal pathways, (2) which is manifested by high production of different cytokines, (3) which disturbs the production of neurotransmitters and induces oxidative damage, with activation of p38 and JNK and decrease in BNDF production. (4) All this causes neuronal injury and death, increases the permeability of the BBB and (5) induces behavioural alterations and neuropsychiatric symptoms such as depression and anxiety. (Created with BioRender.com.).

In conclusion, these results suggest that the activation of immune responses and the release of inflammatory cytokines and afferent nerve stimulus in the lung during pulmonary infection with *M. tb* establish a communication between the inflammatory response within the lung and the CNS, which leads to neuroinflammation in the hypothalamus, hippocampus and cerebellum. The establishment of inflammation within the brain disturbs the levels of the neurotransmitters NE, EP, 5-HT and DA, increases the levels of activated JNK and p38 and the decline in the BDNF levels, which leads to neurodegeneration, neuronal death and BBB dysfunction (Figure 17). All these changes may be related to the behavioural abnormalities present in pulmonary TB and evoke the interest and need to control neuroinflammation in TB patients with neuropsychiatric abnormalities.

## Figures and Tables

**Table 1:** Correlation of behaviour abnormalities with cytokines levels in the hypothalamus, hippocampus and cerebellum showed by TB mice ^1^.

	LMA	TST	SAP	OFT	NSS	STM	LTM
**Hypothalamus**	r	r	r	r	r	r	r
TNFa	0.8833 **	0.7889 *	0.7559 *	−0.07808	0.7317 *	0.1095	0.3309
IL12	0.8184 *	0.5717	0.4409	−0.1747	0.6993	−0.3207	−0.01169
IFNg	0.7815 *	0.6809	0.7112 *	−0.09366	0.6088	0.08314	0.3786
IL4	0.7899 *	0.6198	0.7099 *	0.01956	0.7502 *	−0.2002	0.2053
TGFb	0.6945	0.5908	0.399	0.01386	0.6948	−0.2504	0.177
iNOS	0.02219	0.1924	0.4995	0.3337	0.2651	−0.1323	0.2914
IDO	0.6636	0.4863	0.5386	0.6636	0.6561	−0.3265	0.08468
**Hippocampus**	r	r	r	r	r	r	r
TNFa	−0.1889	0.9552 **	0.9348 *	0.9710 **	0.2837	−0.7992 *	−0.8205 *
IL12	−0.04769	0.8692 *	0.7412 *	0.9112 **	0.4015	−0.7820 *	−0.7183 *
IFNg	0.495	0.631	0.7785 *	0.9417 **	0.4031	0.2029	0.2087
IL4	−0.1975	−0.3987	−0.5243	−0.5828	−0.1602	−0.3492	−0.2862
TGFb	−0.3224	0.05267	−0.4841	0.06559	0.2175	−0.6465	−0.905 **
iNOS	0.4823	0.6369	0.361	−0.1349	0.4144	0.1765	−0.805 **
IDO	0.06284	0.4678	−0.1296	−0.01114	0.4456	−0.3844	−0.5953
**Cerebellum**	r	r	r	r	r	r	r
TNFa	0.8385 **	0.7098 *	0.42	−0.2635	0.6887	−0.2252	0.04278
IL12	0.7584 *	0.7564 *	0.4769	−0.2273	0.5967	0.04006	0.1963
IFNg	0.5856	0.3561	0.1751	−0.1976	0.513	−0.4544	−0.1623
IL4	0.7871 *	0.7646 *	0.4776	−0.2379	0.624	−0.00214	0.1755
TGFb	0.7687 *	0.5827	0.3275	−0.2482	0.647	−0.3513	−0.05443
IL10	−0.3965	−0.03638	−0.3613	−0.2648	−0.4922	0.4757	0.07207
iNOS	0.5856	0.3561	0.1751	−0.1976	0.513	−0.4544	−0.1623
IDO	0.5856	0.3561	0.1751	−0.1976	0.513	−0.4544	−0.1623

^1^ The level of cytokines and enzymes in each brain region was correlated with the behaviour abnormalities scores. Correlations are based on Pearson calculations. The data were first analysed with a Shapiro–Wilk normality test to know the distribution of the data. Once we confirmed that the data had parametric distribution, we performed the Pearson calculations. The media of each expression of cytokine at each time of infection was correlated with the media of each behaviour score at the same time. *p* < 0.05 (two-tailed) * *p* < 0.01, ** *p* < 0.001. LMA (locomotor activity), TST (tail suspension test), SAP (stretched attend posture), OFT (open field test), NSS (neurological severity score) STM (short-term memory) and LTM (long-term memory). See Supplemental Table S2 for exact *p* values for all comparisons made during the post-hoc test.

## Supplementary Materials

Supplementary Materials can be found at https://www.mdpi.com/1422-0067/21/24/9483/s1.

## Abbreviations

ANOVA	Analysis of variance
BCG	Bacillus Calmette–Guerin
BSL-3	Biosafety laboratory (level three)
BBB BSA CSF CNS CVOs JNK CFU COX-2 DG DA EP EDTA EB ERK FJ-B GAPDH GM-CSF HPLC HPA IDO iNOS IFNα IFNγ IL i.p. LMA LOD LOQ MAPK MCP-1 MPA MPB *M. tb* NSS BDNF NE NFκB NTS OADC PD PAMPS PMSF TB RT–PCR 5-HT SEM SAP TLR TGFβ TNFα Th1 Th2 uPA VEGF	Blood–brain barrier Bovine serum albumin Cerebrospinal fluid Central nervous system Circumventricular organs c-Jun NH2-terminal kinase Colony forming units Cyclooxygenase-2 Dentate gyrus Dopamine Epinephrine Ethylenediaminetetraacetic acid Evans blue Extracellular signal-regulated kinase Fluoro-Jade B Glyceraldehyde-3-phosphate dehydrogenase Granulocyte-macrophage colony-stimulating factor High-performance liquid chromatography Hypothalamic–pituitary–adrenal axis Indolamine 2,3-dioxygenase Induced nitric oxide synthase Interferon-alpha Interferon-gamma Interleukin Intraperitoneal Locomotor activity Limit of detection Limit of quantification Mitogen-activated protein kinases Monocyte chemoattractant protein-1 Mobile phase A Mobile phase B Mycobacterium tuberculosis Neurological severity score Neurotrophin brain-derived nerve growth factor Norepinephrine Nuclear factor kappa B Nucleus tractus solitarius Oleic acid, albumin, dextrose and catalase Pixel densities Pathogen-associated molecular patterns Phenylmethylsulfonyl fluoride Tuberculosis Reverse transcription-polymerase chain reaction Serotonin Standard error of the mean Stretched attend posture Toll-like receptor Transforming growth factor type-beta Tumour necrosis factor-alpha Type 1 cooperating lymphocytes Type 2 Cooperating lymphocytes Urokinase-type plasminogen activator Vascular endothelial growth factor
