# Supplementary material for: Experimental Pulmonary Tuberculosis in the Absence of Detectable Brain Infection Induces Neuroinflammation and Behavioural Abnormalities in Male BALB/c Mice
Source: Int J Mol Sci. 2020 Dec 13;21(24):9483. doi: 10.3390/ijms21249483 (PMC7763936; doi:10.3390/ijms21249483)
Supplement: Supplementary file 1 [file ijms-21-09483-s001.zip › Supplementary Files/Supplemental Table 1.docx]

**Supplemental Table 1.** Neurological Severity Score (NSS) for mice. A 31-point neurological severity score (NSS) was used for assessment of neurological impairment. This score is derived from a 10-point NSS, which was previously used for assessment of neurological impairment [70].

| **Task** | **Description** | Points | |
| --- | --- | --- | --- |
|  |  | Success | Failure |
| Hypomobility | Lower spontaneous mobility of the experimental mouse as compared with that of the external control when placed (30 s) on the top of the table. Scored 1,2 or 3 regarding locomotion, and speed and vigors of movements. | 0 | 1,2,3 |
| Lateralised posture | Abnormal body position characterised by a persistent tendency to recline sideward | 0 | 1 |
| Flattened posture | Abnormal body position characterised by slow movements and dragging the body along the tabletop | 0 | 1 |
| Hunched back | The persistent presence of a crouched posture | 0 | 1 |
| Piloerection | Persistent rise of the back hair. | 0 | 1 |
| Ataxic gait | The tendency to sway, rock, or lunch to the side as the animal proceeds forward. | 0 | 1 |
| Circling | Spontaneous or forced (gently pushing with one finger) walking consistently to one side. | 0 | 1 |
| Tremors | Presence of subtle, repetitive, oscillatory movements observed during movement. | 0 | 1 |
| Twitches | Abrupt body jerks. | 0 | 1 |
| Convulsions | Repetitive twitches followed by extensions of the hind limbs. | 0 | 1 |
| Respiratory distress | Presence of increased, irregular, respiratory movements accompanied by breathing sounds. | 0 | 1 |
| Passivity | Characterised by the decreased behavioural response (struggle and escape) when the animals are covered with the hand to restrain movement. | 0 | 1 |
| Hyperreactivity | Characterised by the exaggerated behavioural response (struggle and escape) when the animals are covered with a hand to restrain movement. | 0 | 1 |
| Irritability | Characterised by aggressive posture and bitting behaviour exhibited by the animals when covered with a hand to restrain movement. | 0 | 1 |
| Ptosis | Closure of dropping of the upper eyes. | 0 | 1 |
| Urination | An excessive amount of urine on the animal´s body and the tabletop | 0 | 1 |
| Decreased body tone | Characterised by relatively less resistance to compression or flaccidity of the abdominal muscles, determined by gentle compression of the sides of the animal between the lower thorax and pelvis using the thumb and index finger. | 0 | 1 |
| Forelimb flexion | Failure to extend one forepaw fully when the animal is held by the tail to a height of 10 cm and slowly lowered to observe symmetry in the outstretching of both forelimbs while the mouse reached the wire-mesh. | 0 | 1 |
| Decreased muscle strength | Characterised by decrease resistance when the animal is placed off the grid and gently drawn backwards by the tail. | 0 | 1 |
| Body rotation | Rolling along the axis of its body when the animal is held by the tail. | 0 | 1 |
| Motor incoordination | Characterised by decrease capacity to move and remain for at least 10 sec on the inclined plane (45°) or to grasp the cord (30 cm above the tabletop) with limbs and tail, and to remain there for a least 10 sec. | 0 | 1,2,3 |
| Absence of equilibrium | Characterised by a decreased capacity to remain for at least 10 sec on a horizontal bar (1 cm wide). Four paws have to be on the bar. | 0 | 1,2,3 |
| Hypoalgesia | Lack of behavioural response when an arterial claw is placed at 1 cm from the base of the animal´s tail. | 0 | 1 |
| Hyperalgesia | Exaggerated behavioural response (disproportional vocalisation and bitting) when an arterial claw is placed at 1 cm from the base of the animal´s tail. | 0 | 1 |
| Sensitivity in the whiskers | Behavioural response when animal´s moustaches are touched with a hyssop | 0 | 1 |
| Sensitivity in the abdomen | Behavioural response when the animal in gently touched in the abdomen. | 0 | 1 |
| Olfactory sensitivity | Behavioural response when hyssop with alcohol is placed in the noise of the animal | 0 | 1 |
| **Global Score** | | | **31** |
