# Supplementary material for: Experimental Pulmonary Tuberculosis in the Absence of Detectable Brain Infection Induces Neuroinflammation and Behavioural Abnormalities in Male BALB/c Mice
Source: Int J Mol Sci. 2020 Dec 13;21(24):9483. doi: 10.3390/ijms21249483 (PMC7763936; doi:10.3390/ijms21249483)
Supplement: Supplementary file 1 [file ijms-21-09483-s001.zip › Supplementary Files/Supplemental Table 2. Exact p values for Correlation analysis .docx]

**Supplemental Table 2. Exact p values for Correlation analysis**

|  | **LMA** | **TST** | **SAP** | **OFT** | **NSS** | **STM** | **LTM** |
| --- | --- | --- | --- | --- | --- | --- | --- |
| **Hypothalamus** | **P value** | **P value** | **P value** | **P value** | **P value** | **P value** | **P value** |
| TNFα | ***0.0036^**^*** | ***0.0199^*^*** | ***0.0300^*^*** | 0.8542^ns^ | ***0.0391^*^*** | 0.7964^ns^ | 0.4234^ns^ |
| IL12 | ***0.0130**** | 0.1387^ns^ | 0.4409 | 0.6790^ns^ | 0.0536^ns^ | 0.4386^ns^ | 0.9781^ns^ |
| IFNγ | ***0.0220**** | 0.0630^ns^ | ***0.0479**** | 0.8254^ns^ | 0.1092^ns^ | 0.8448^*^ | 0.3550^ns^ |
| IL4 | **0.0197*** | 0.1012^ns^ | ***0.0485**** | 0.9633^ns^ | ***0.0320**** | 0.6346 | 0.6257^ns^ |
| TGFβ | 0.0560^ns^ | 0.1230^ns^ | 0.0929^ns^ | 0.9740^ns^ | 0.0558^ns^ | 0.5497^ns^ | 0.6749^ns^ |
| iNOS | 0.9584^ns^ | 0.6480^ns^ | 0.2076^ns^ | 0.4192^ns^ | 0.5257^ns^ | 0.7549^ns^ | 0.4838 |
| IDO | 0.0728^ns^ | 0.2217^ns^ | 0.1684^ns^ | 0.0728^ns^ | 0.0772^ns^ | 0.4299^ns^ | 0.8420^ns^ |
| **Hippocampus** | **P value** | **P value** | **P value** | **P value** | **P value** | **P value** | **P value** |
| TNFα | 0.6542^ns^ | **0.0023**** | **0.0120*** | **0.0038**** | 0.4960^ns^ | **0.0136*** | **0.0126*** |
| IL12 | 0.9107^ns^ | **0.0214*** | **0.0124*** | **0.0024**** | 0.3242^ns^ | **0.0205*** | **0.0447*** |
| IFNγ | 0.2124^ns^ | 0.0934^ns^ | **0.0305*** | **0.0012**** | 0.3221^ns^ | 0.6299^ns^ | 0.6198^ns^ |
| IL4 | 0.6392^ns^ | 0.3279^ns^ | 0.1822^ns^ | 0.1295^ns^ | 0.7046^ns^ | 0.3965^ns^ | 0.4920^ns^ |
| TGFβ | 0.4361^ns^ | 0.9014^ns^ | 0.2242^ns^ | 0.8774^ns^ | 0.6048^ns^ | 0.0832^ns^ | **0.0020**** |
| iNOS | 0.2261 | 0.0894^ns^ | 0.3796^ns^ | 0.7500^ns^ | 0.3073^ns^ | 0.6759^ns^ | **0.0031**** |
| IDO | 0.8825^ns^ | 0.2424^ns^ | 0.7597^ns^ | 0.9791^ns^ | 0.2685^ns^ | 0.3471^ns^ | 0.1195^ns^ |
| **Cerebellum** | **P value** | **P value** | **P value** | **P value** | **P value** | **P value** | **P value** |
| TNFα | ***0.0093***** | ***0.0486**** | 0.3002^ns^ | 0.5283^ns^ | 0.0589^ns^ | 0.5918^ns^ | 0.9199^ns^ |
| IL12 | ***0.0292**** | ***0.0298**** | 0.2321^ns^ | 0.5883^ns^ | 0.1184^ns^ | 0.9250^ns^ | 0.6413^ns^ |
| IFNγ | 0.1272^ns^ | 0.3865^ns^ | 0.6783^ns^ | 0.6391^ns^ | 0.1935^ns^ | 0.2580^ns^ | 0.7011^ns^ |
| IL4 | ***0.0204**** | ***0.0271**** | 0.2314^ns^ | 0.5705^ns^ | 0.0982^ns^ | 0.9960^ns^ | 0.6777^ns^ |
| TGFβ | ***0.0258**** | 0.1296^ns^ | 0.4285^ns^ | 0.5534^ns^ | 0.0829^ns^ | 0.3935^ns^ | 0.8981^ns^ |
| IL10 | 0.3308^ns^ | 0.9318 | 0.3793^ns^ | 0.5262^ns^ | 0.2153^ns^ | 0.2335^ns^ | 0.8653^ns^ |
| iNOS | 0.1272^ns^ | 0.3865^ns^ | 0.6783^ns^ | 0.6391^ns^ | 0.1935^ns^ | 0.2580^ns^ | 0.7011^ns^ |
| IDO | 0.1272^ns^ | 0.3865^ns^ | 0.6783^ns^ | 0.6391^ns^ | 0.1935^ns^ | 0.2580^ns^ | 0.7011^ns^ |
